# How Diverse Are the Protein-Bound Conformations of Small-Molecule Drugs and Cofactors?

**DOI:** 10.3389/fchem.2018.00068

**Published:** 2018-03-27

**Authors:** Nils-Ole Friedrich, Méliné Simsir, Johannes Kirchmair

**Affiliations:** ^1^Department of Informatics, Center for Bioinformatics, Universität Hamburg, Hamburg, Germany; ^2^Molécules Thérapeutiques In Silico, Université Paris Diderot, Sorbonne Paris Cité, Paris, France

**Keywords:** bioactive conformational space, protein-bound ligand conformation, conformational variability, PDB, protein-ligand interaction, binding site, small-molecule drug, cofactor

## Abstract

Knowledge of the bioactive conformations of small molecules or the ability to predict them with theoretical methods is of key importance to the design of bioactive compounds such as drugs, agrochemicals, and cosmetics. Using an elaborate cheminformatics pipeline, which also evaluates the support of individual atom coordinates by the measured electron density, we compiled a complete set (“Sperrylite Dataset”) of high-quality structures of protein-bound ligand conformations from the PDB. The Sperrylite Dataset consists of a total of 10,936 high-quality structures of 4,548 unique ligands. Based on this dataset, we assessed the variability of the bioactive conformations of 91 small molecules—each represented by a minimum of ten structures—and found it to be largely independent of the number of rotatable bonds. Sixty-nine molecules had at least two distinct conformations (defined by an RMSD greater than 1 Å). For a representative subset of 17 approved drugs and cofactors we observed a clear trend for the formation of few clusters of highly similar conformers. Even for proteins that share a very low sequence identity, ligands were regularly found to adopt similar conformations. For cofactors, a clear trend for extended conformations was measured, although in few cases also coiled conformers were observed. The Sperrylite Dataset is available for download from http://www.zbh.uni-hamburg.de/sperrylite_dataset.

## Introduction

The protein-bound (“bioactive”) conformations of ligands can differ substantially from those observed in solution, the gas phase and small-molecule crystal structures (Boström, 2001; Perola and Charifson, 2004; Seeliger and de Groot, 2010). Bioactive conformations can be distributed over large regions of the ligand's conformational space and can have considerable strain energy (Nicklaus et al., 1995; Boström et al., 1998; Boström, 2001; Perola and Charifson, 2004; Günther et al., 2006). For the application of 3D computational approaches such as docking or *de novo* design methods in drug discovery, the protein-bound conformations of small molecules need to be known or at least determinable (Brameld et al., 2008).

The Protein Data Bank (PDB) is the most comprehensive resource of experimental structural data on biomacromolecules and their interaction with small molecules (Berman et al., 2000). Currently, the PDB contains more than 100k structures of biomacromolecules that include a bound ligand. While the structural data available from the PDB are extremely valuable for the research of biomacromolecules and their interactions with small molecules, these data represent only a very small fraction of (known) interactions.

Sturm et al. (2012) investigated the relationship between the promiscuity of drug-like molecules and the molecular properties of ligands and their binding sites. In order to do so, they compiled a dataset of more than 1,000 protein-ligand complexes in which drug-like molecules are bound to at least two distinct proteins. They identified two major drivers of ligand promiscuity: the structural similarities of ligand binding sites (largely independent of the similarities of the overall protein sequences or folds) and the ability of ligands to adopt distinct binding modes for different proteins. The latter is facilitated by the conformational flexibility of ligands and/or the specific characteristics of their pharmacophoric features. In related work, He et al. (2015), analyzed the structures of 100 pharmaceutically relevant ligands bound to at least two different proteins (to which they bind with comparable *in vitro* affinities). Contrary to the common belief that ligand flexibility and promiscuity are correlated, no evidence for a distinct correlation was found within their dataset. In fact, for 59 out of the 100 investigated ligands, no significant changes between the conformers of ligands bound to different proteins were observed.

The relative abundance of available structural data on the conformation of protein-bound cofactors, and nucleotide cofactors in particular, has made them a primary subject of investigation. For example, Moodie and Thornton (1993) analyzed 65 structures of nucleotides bound to proteins and found them to bind predominantly in an extended conformation. In more recent work, Stockwell and Thornton (2006) analyzed the conformational variability of adenosine triphosphate (ATP), nicotinamide adenine dinucleotide (NAD) and flavin adenine dinucleotide (FAD) in a preprocessed set of more than 2,000 structures extracted from the PDB. Dym and Eisenberg (2001) compiled a set of 150 structures of FAD bound to 32 non-redundant flavoproteins. They found a clear correlation between the FAD-family fold, the shape of the cofactor binding site and the conformation of FAD. Bojovschi et al. (2012) investigated the conformational diversity of ATP/Mg:ATP in motor proteins based on a set of 159 X-ray structures extracted from the PDB. They found that ATP adopts a wide range of different conformations, with a preference for extended conformations in tight binding pockets (e.g., F1-ATPase) and compact conformations in motor proteins such as RNA polymerase and DNA helicase. The incorporation of Mg2+ was found to increase the conformational flexibility of ATP. They clustered the conformations of the individual ligands based on the similarity of their binding pockets and, in the case of ATP for example, identified 27 clusters with a mean intercentroid RMSD of more than 2 Å. The authors concluded that, within the individual protein superfamilies, the investigated ligands generally bind in a fairly conserved manner, although several exceptions were identified. In the case of ATP, most structures were found to have the ligand bound in an extended conformation. In few cases however, a conformation bent such that the terminal phosphate atoms are almost in van der Waals contact with the adenine ring was observed. Stegemann and Klebe (2012) explored the structural properties of six cofactors including an adenosine diphosphate moiety bound to a variety of different proteins with low sequence identity. They found that common binding pocket patterns sometimes only recognize parts of the cofactor and thereby induce similar conformations.

These and further studies have contributed substantially to the understanding of protein-bound ligand conformations. However, a major bottleneck is the limited quality (Liebeschuetz et al., 2012; Reynolds, 2014), quantity and diversity of the structural data that these studies are based on, in particular with respect to the uncertainty of atom coordinates that is inherent to crystallographic structures. Only recently, a robust and fully automated method for the assessment of the support of individual atom coordinates (as well as molecules) by the measured electron density (EDIA) has become available (Meyder et al., 2017). This allowed, for the first time, extraction of a complete subset of high-quality structures of protein-bound ligands from the PDB (Friedrich et al., 2017b). Prior to the development of the EDIA method, time-consuming manual inspection by human experts was required to assure the high quality of structural data, which limited the size of available datasets (see e.g., Warren et al., 2012).

In this work we assess the conformational variability of small molecules based on a complete set of high-quality structures of protein-bound ligands extracted from in the PDB, each of which is represented by at least ten high-quality X-ray structures. In total the conformational variability of 91 approved drugs and cofactors represented by 4,574 protein-bound conformations was assessed. The bioactive conformational space of 17 representative molecules was studied in detail.

## Materials and methods

### Dataset compilation

The Sperrylite Dataset was extracted from the PDB using a workflow described previously (Friedrich et al., 2017a). It consists of 10,936 conformers of 4,548 unique small molecules. Ninety-one ligands in this dataset are represented by at least 10 structures, and these served as the basis of this analysis.

To ensure that all ligands with the same PDB ligand ID have identical stereochemistry, their isomeric smiles (generated with UNICON, Sommer et al., 2016) were compared in order to keep only the isomer with the most occurrences. The Approved Drugs subset of DrugBank (Wishart et al., 2017) was used to identify the approved drugs present in the Sperrylite Dataset.

### RMSD, rotatable bonds and sequence identity calculations

All RMSD values were calculated with NAOMI (Urbaczek et al., 2011), which selects the minimum heavy-atom RMSD for the best superposition of each pair of conformers, taking molecular symmetry into account via complete automorphism enumeration.

The number of rotatable bonds was calculated with RDKit (RDKit: Open-Source Cheminformatics, version 2015.09.1, 2015). The default definition was used, meaning that amide and ester bonds were not counted as rotatable bonds.

All-against-all sequence identity was determined with NCBI BLAST (Altschul et al., 1990; BLAST, version 2.2.31. https://blast.ncbi.nlm.nih.gov (accessed Jan 14, 2018); Camacho et al., 2009) and the sequence identity of individual pairs of proteins was measured with the Molecular Operating Environment (Molecular Operating Environment (MOE), version 2016.08; Chemical Computing Group Inc.: Montreal, QC, 2016) based on sequence and structural alignments.

Principal component analysis (PCA)-derived score plots of the alignments with the minimum median RMSDs were generated with R for each ligand.

### Visualization

Visualization of the (i) alignments of ligand conformers (ii) alignments of protein structures and (iii) interactions of proteins and ligands were generated with Maestro (Schrödinger Release 2016-2: Maestro, Schrödinger, LLC, New York, NY, 2016), MOE (Molecular Operating Environment (MOE), version 2016.08; Chemical Computing Group Inc.: Montreal, QC, 2016) and LigandScout (LigandScout, version 4.2; Inte:Ligand GmbH: Vienna, Austria, 2017; Wolber and Langer, 2005), respectively.

For the sake of clarity, all hydrogens, only polar hydrogens or no hydrogens were included in the depictions on a case-by-case basis to avoid overcrowded figures.

## Results

The Sperrylite Dataset is a collection of all high-quality X-ray structures of small molecules bound to biomacromolecules that are contained in the PDB. The dataset includes 10,936 structures of 4,548 unique protein-bound ligands and was compiled with a recently developed cheminformatics pipeline that automatically (i) prepares the chemical structures of small molecules by taking into account the protein environment (in order to determine, e.g., the most likely tautomeric and protonation states); (ii) removes undesirable molecules such as crystallization aids as well as structures with topological and/or geometrical errors; and (iii) rejects structures of low quality (Friedrich et al., 2017a,b). Importantly, the procedure not only includes checks for resolution and DPI (Cruickshank, 1999), but also employs the recently developed EDIA method (Meyder et al., 2017) to assess the support of individual atoms of a structure by the electron density.

In this study the diversity of the protein-bound conformations of all ligands represented by at least 10 high-quality structures was investigated. This dataset consists of a total of 4,574 conformations of 91 unique ligands (an overview of all structures is provided in Scheme S1), including more than 30 nucleotides and 20 approved drug molecules. In an all-against-all comparison of the differences in conformation of each ligand as measured by RMSD, 81 of the 91 ligands had at least one conformer with an RMSD above 0.6 Å (which corresponds to the maximum positional uncertainty for atoms in the Sperrylite Dataset), and 69 had at least one conformer above 1 Å, meaning that they are clearly distinct. The correlation observed between the minimum median RMSD measured for all pairs of conformations and the number of rotatable bonds was (very) weak (*R*^2^ = 0.126; Figure S1).

This work focuses on the analysis of the bioactive conformational space of a representative set of 17 approved drugs and cofactors (Tables 1, 2; note that there is an overlap between cofactors and approved drugs). This set was compiled with the objective to include the most relevant and best-represented small molecules in a detailed analysis of individual ligands.

**Table 1:** Summary of approved drugs investigated in this work.

**Name**	**No. of PDB entries**	**Protein names: No. of high-quality conformers**	**No. of Confs.^a^**	**Major observations**
Imatinib (STI)	18	Tyrosine kinases: 10 Quinone reductase 2: 1	2	Conformers for different tyrosine kinases are similar, even for pairs of proteins with low sequence identity. A distinct conformation is observed in a complex with quinone reductase 2
Darunavir (017)	54	HIV-1 protease: 14	1	Conformers are highly similar, also those in complex with various different mutants of this protein
Acetazolamide (AZM)	29	Carboanhydrases: 9 Endochitinase: 1	n.d.^b^	It is likely that the ligand binds in a similar conformation to all proteins covered by the dataset (the experimental data do not allow a definitive conclusion)
Triclosan (TCL)	31	Enoyl-acyl carrier protein reductases: 11	1	All conformers are highly similar. The median RMSD is 0.1 Å and the maximum pairwise RMSD is below 0.6 Å
Ubenimex/bestatin (BES)	28	Aminopeptidases: 9 Leukotriene A-4 hydrolase: 2	3	Conformations observed for most (even distantly) related aminopeptidases and human leukotriene A-4 hydrolases are similar, with the exception of one conformation observed in complex with human aminopeptidase N
Biotin (BTN)	99	Streptavidin: 24 Avidin: 7 Biotin-protein ligase: 6 Others: 6	3	Conformations observed among the different complexes with core streptavidin are very similar. Two distinct conformers are observed in complex with biotin-protein ligase and biotin carboxylase
Sapropterin (H4B)	472	Total: 188	2	All but three conformers are extremely similar to each other (median RMSD smaller than 0.1 Å), even for distantly related proteins
Cholic acid (CHD)	74	Cytochrome c oxidase: 2 Ferrochelatase: 2 Alcohol dehydrogenase: 1 Others: 8	4	Due to the rigid steroid scaffold, the conformations observed for both ligands are all highly similar
Deoxycholic acid (DXC)	29	Cathepsin A: 11 Bet v1: 2 Others: 3	2	

a *No. of distinct bioactive conformations*.

b *The experimental data are insufficient to allow a definitive conclusion on the number of distinct bioactive conformations*.

**Table 2:** Summary of cofactors and cofactor analogs investigated in this work.

**Name**	**No. of PDB entries**	**Protein names: No. of high-quality conformers**	**No. of Confs.^a^**	**Major observations**
Sinefungin (SFG)	70	Methyltransferase: 23 Others: 7	6	Three clusters of conformers are observed. The largest group includes 23 highly similar conformers and includes structures bound to proteins that share low sequence identity. The maximum RMSD measured for any of the sinefungin conformers is 3.6 Å
S-adenosylmethionine (SAM)	410	Methyltransferase: 92 Others: 31	24	A wide variety of conformations are observed, with a clear clustering into three distinct groups of conformers. Within these groups, a large number of similar conformers are observed, even when bound to proteins sharing low sequence identity. The maximum pairwise RMSD is 3.3 Å
S-Adenosyl-L-Homocysteine (SAH)	784	Methyltransferase: 284 RNA polymerase: 8 Others: 19	23	
Glutathione (GSH)	360	Glutathione transferase: 46 Others: 28	16	Most of the conformers have a pairwise RMSD between 0.6 and 1.6 Å, but the maximum pairwise RMSD is 3.6 Å
Adenosine monophosphate (AMP)	575	Total: 171	36	A wide variety of different conformers is observed. One distinct, extremely coiled conformer was observed in complex with an adenylate kinase-related protein. The maximum pairwise RMSD is 2.5 Å
Adenosine diphosphate (ADP)	1,810	Total: 462	81	The conformers are similar to those observed for AMP, despite the presence of an additional phosphate group. The median RMSD is 0.9 Å
Adenosine triphosphate (ATP)	1,079	Total: 218	76	ATP is observed in an extended conformation in most structures, but some conformers are extremely bent. The median and the maximum pairwise RMSDs are 1.6 and 3.9 Å, respectively
Flavin mononucleotide (FMN)	919	Total: 367	21	The overall median RMSD is 0.9. The all-against-all comparison revealed four groups of conformers, with peaks in the RMSD distribution at around 0.3, 1.2, 1.7, and 2.4 Å

a *No. of distinct bioactive conformations*.

### Definitions

In the following sections, “high-quality structures” refers to any structures matching the quality criteria defined in previous work (Friedrich et al., 2017b). Importantly, this term only refers to the quality of the protein-bound ligand, not the overall structure of the protein-ligand complex. Four-letter codes refer to PDB entries and three-letter codes in italics refer to PDB ligand identifiers.

### Small-molecule drugs

#### Imatinib

Imatinib (*STI*) is an approved anti-cancer drug targeting Bcr-Abl and several other tyrosine kinases. The drug binds to the ATP-binding site, spanning almost the entire width of the protein (Reddy and Aggarwal, 2012). Imatinib locks the protein in a closed conformation, thus arresting the enzyme's functionality. The PDB lists 11 high-quality structures with imatinib, 10 thereof with the drug bound to one of three different tyrosine kinases (ABL1: 1IEP, 1OPJ, 3K5V, 3MS9, 3MSS, 3PYY; ABL2: 3GVU; c-Src: 2OIQ, 3OEZ) or a synthetic construct of tyrosine kinase AS (4CSV), a common ancestor of Src and Abl.

**Figure 1:**
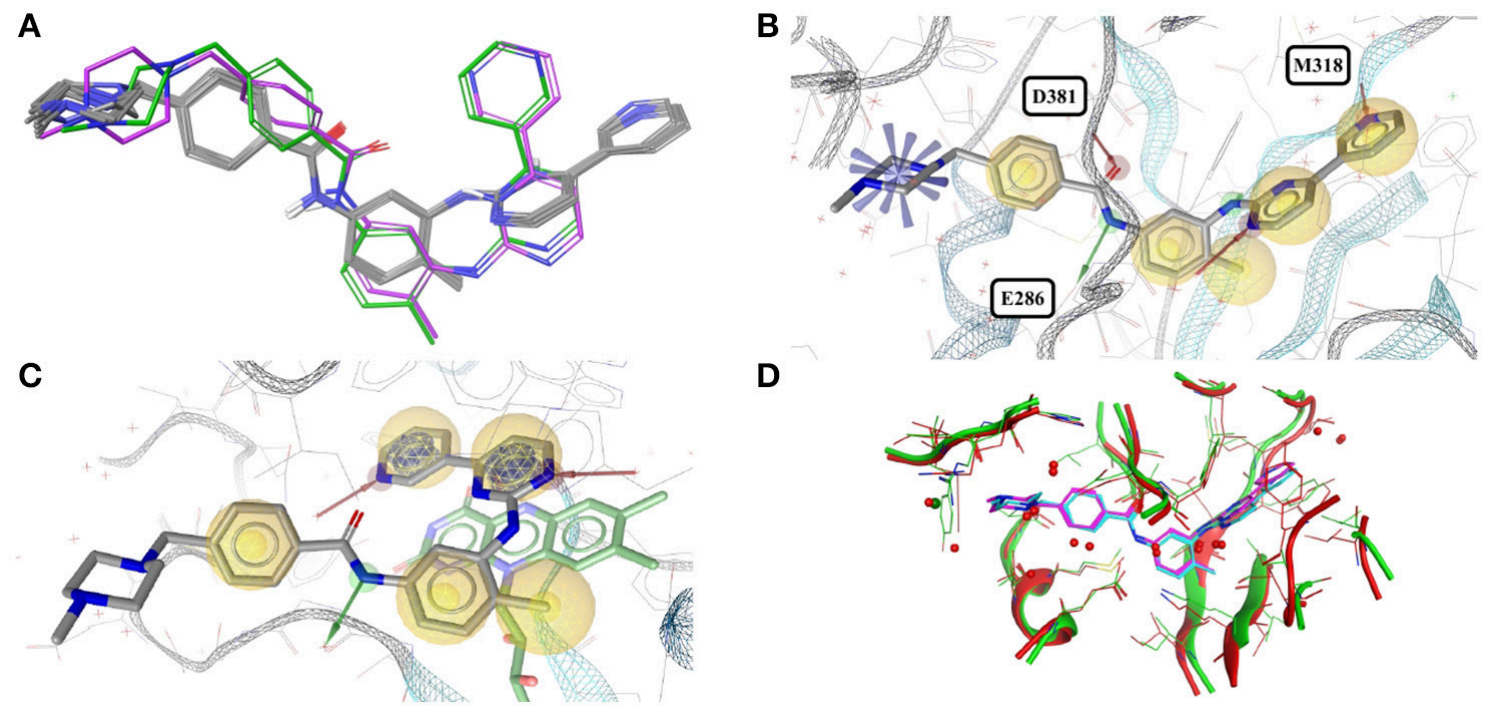
**(A)** Ligand-based alignment of imatinib conformers observed in complex with three different tyrosine kinases (gray carbon atoms), human quinone reductase 2 (3FW1; violet carbon atoms) and human spleen tyrosine kinase (1XBB; green carbon atoms). **(B)** Imatinib bound to ABL1 (3MS9) in an extended conformation that is characteristic for the drug bound to tyrosine kinases. Red and green vectors indicate hydrogen bond donors and acceptors, respectively. Yellow spheres mark hydrophobic moieties involved in interactions with the protein, and blue astral centers indicate charge interactions involving a positively charged group on the ligand side. **(C)** Imatinib bound to human quinone reductase 2 in a conformation that is different from those characteristic of tyrosine kinases (3FW1; FAD with green carbon atoms). **(D)** Alignment of the binding sites of human ABL1 (3K5V; red) and c-Src (2OIQ; green). Despite a sequence identity of only 45%, the ligand binding sites of both proteins are almost identical.

The accessible conformational space of imatinib, which has seven rotatable bonds, is large. However, the conformations observed for imatinib bound to any of these tyrosine kinases are similar (Figures 1A,B), which is reflected by the low maximum pairwise RMSD of just 0.3 Å and is in agreement with the findings of He et al. (2015). This conformational similarity can be explained by the highly conserved nature of the residues that form the ligand binding sites of these tyrosine kinases (the minimum pairwise sequence identity between these proteins is 45%; Figure 1D).

One high-quality structure of imatinib is a complex with human quinone reductase 2 (3FW1). This enzyme exists as a dimer with two active sites, each located in a deep pocket at the interface between the monomers (Foster et al., 1999; Winger et al., 2009). Quinone reductase 2 is structurally dissimilar to protein kinases. Imatinib binds to the enzyme active site in proximity to the isoalloxazine ring of the FAD cofactor (Figure 1C), thereby adopting a distinct, “horseshoe-like” conformation (Winger et al., 2009) that differs by at least 2.4 Å from any of the conformations observed with tyrosine kinases (Figure 1A).

Note that imatinib is known to bind to spleen tyrosine kinase (SYK) in an orientation that is different from that observed for Bcr-Abl and other tyrosine kinases (Alton and Lunney, 2008). A crystal structure of the imatinib-SYK complex exists (1XBB; Atwell et al., 2004) but is not part of the Sperrylite Dataset because of a poor electron density support of parts of the ligand facing the bulk water phase (Figure S2). The conformer of imatinib in complex with SYK has an RMSD of 2.5 Å to any of the other kinase-bound conformers but is similar to the imatinib conformation observed in the complex with quinone reductase 2 (RMSD = 1.3 Å).

#### Darunavir

Darunavir (*017*) is an antiretroviral drug approved for the treatment and prevention of human immunodeficiency virus (HIV) infections. The compound inhibits HIV-1 protease at picomolar concentrations by forming strong polar interactions with the target enzyme (King et al., 2004). Fourteen out of the 54 available structures with darunavir are of high quality, all of them being structures with darunavir bound to wild type or mutant HIV-1 protease. The mutations observed in the 14 high-quality structures introduce only subtle changes to the shape and chemical properties of the ligand binding environment. This is reflected in the high similarity of the protein-bound conformations of darunavir, where, among the high-quality structures, a maximum pairwise RMSD of just 0.2 Å was measured (Figure S3).

#### Acetazolamide

**Figure 2:**
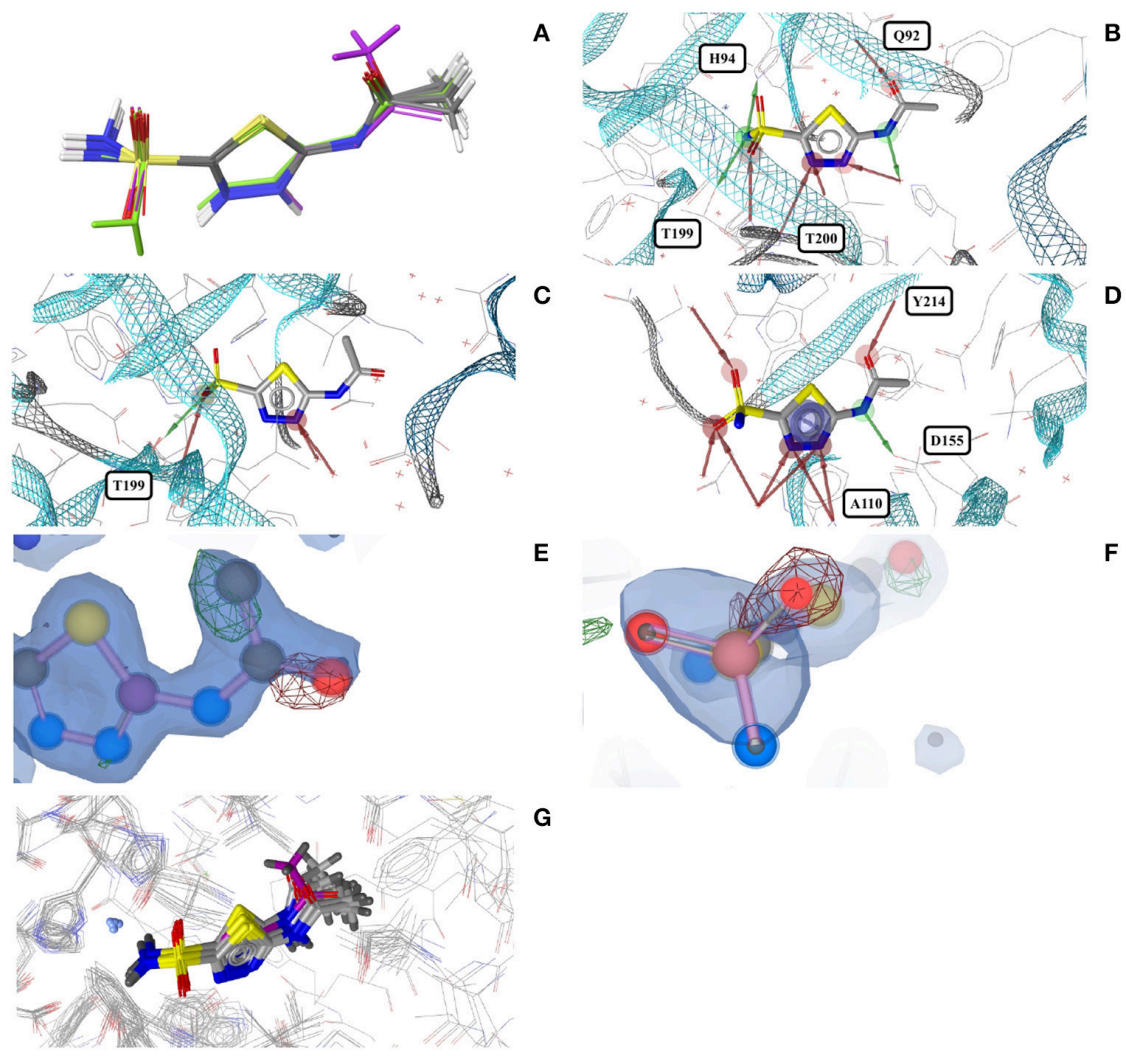
**(A)** Ligand-based alignment of acetazolamide bound to different carbonic anhydrases (gray carbon atoms, except those of 1JD0, which are violet) and endochitinase (2UY4; green). **(B)** The acetyl group of acetazolamide forms hydrogen bond interactions with some carbonic anhydrases such as isozyme VII (3ML5) depicted here. **(C)** In a complex with human carbonic anhydrase XII (1JD0) the acetyl group of acetazolamide is rotated by about 140°. **(D)** In a complex with endochitinase (2UY4), the sulfonamide moiety of acetazolamide is rotated by about 120°. The support of atom positions by the measured electron density can be quantified by the EDIA score. For some of the atoms of the acetyl **(E)** and sulfonamide groups **(F)** of these structures the EDIA scores are below 0.8, meaning that their exact position is uncertain. The 2Fo-Fc, Fo-Fc(–ve) and Fo-Fc(+ve) sigma maps are shown in blue, red and green, respectively. It can therefore not be excluded that the acetyl group in **(C)** and the sulfonamide moiety in **(D)** are present in the same orientation that is observed in any of the other crystal structures. **(G)** Superposed binding pockets of the nine human and three extremophilic bacterial carbonic anhydrases.

Acetazolamide (*AZM*) is an inhibitor of carbonic anhydrase and approved for the treatment of glaucoma, cardiac edema, idiopathic intracranial hypertension, epilepsy, and altitude sickness (Chakravarty and Kannan, 1994; Kaur et al., 2002). Ten out of the 29 structures of acetazolamide listed in the PDB are of high quality. Nine of these structures are with acetazolamide bound to one of six different human carbonic anhydrases (isoforms II, VII, IX, XII, XIII, and XIV, represented by PDB entries 3V2J, 3ML5, 3IAI, 1JD0, 3CZV, and 4LU3, respectively) or three different extremophilic bacteria carbonic anhydrases (*Sulfurihydrogenibium* sp., *Thermovibrio ammonificans*, and *Sulfurihydrogenibium azorense*, represented by PDB entries 4G7A, 4UOV, and 4X5S, respectively). The ligand binding pockets of all these carbonic anhydrase isozymes are highly similar (Figure 2G) and so are the conformations of acetazolamide observed for these complexes (Figure 2A). The protein-ligand complexes are stabilized by hydrogen bonds formed between the acetyl group of acetazolamide and the binding pocket (Figure 2B), with one exception, which is a complex with human carbonic anhydrase XII (1JD0). In that structure, the acetyl group of the ligand is rotated by about 140° as compared to any of the other structures (RMSD 0.9 Å; Figure 2C). A second, distinct conformation of acetazolamide is found in a complex with a different enzyme, endochitinase from *Saccharomyces cerevisiae* (2UY4) with a fundamentally different binding pocket. In that structure, the carbon-sulfur bond of the ligand is rotated by 120° (Figure 2D). The moieties in question are oriented toward the bulk water phase, freely rotatable, and not engaged in directed interactions with the protein. Also, the electron density maps do not allow a definitive conclusion on the orientation of these moieties (Figures 2E,F). It is therefore entirely possible that in reality all conformers of acetazolamide in the Sperrylite Dataset are nearly identical.

#### Triclosan

Triclosan (*TCL*) is an antibacterial and antifungal agent inhibiting enoyl-acyl carrier protein reductases (ENR), which are key enzymes in the fatty acid elongation cycle. Its wide use as a disinfectant in cremes and consumer products (e.g., soaps, toothpaste, detergents) is a controversial topic nowadays (Buth et al., 2010; Carey and McNamara, 2014).

**Figure 3:**
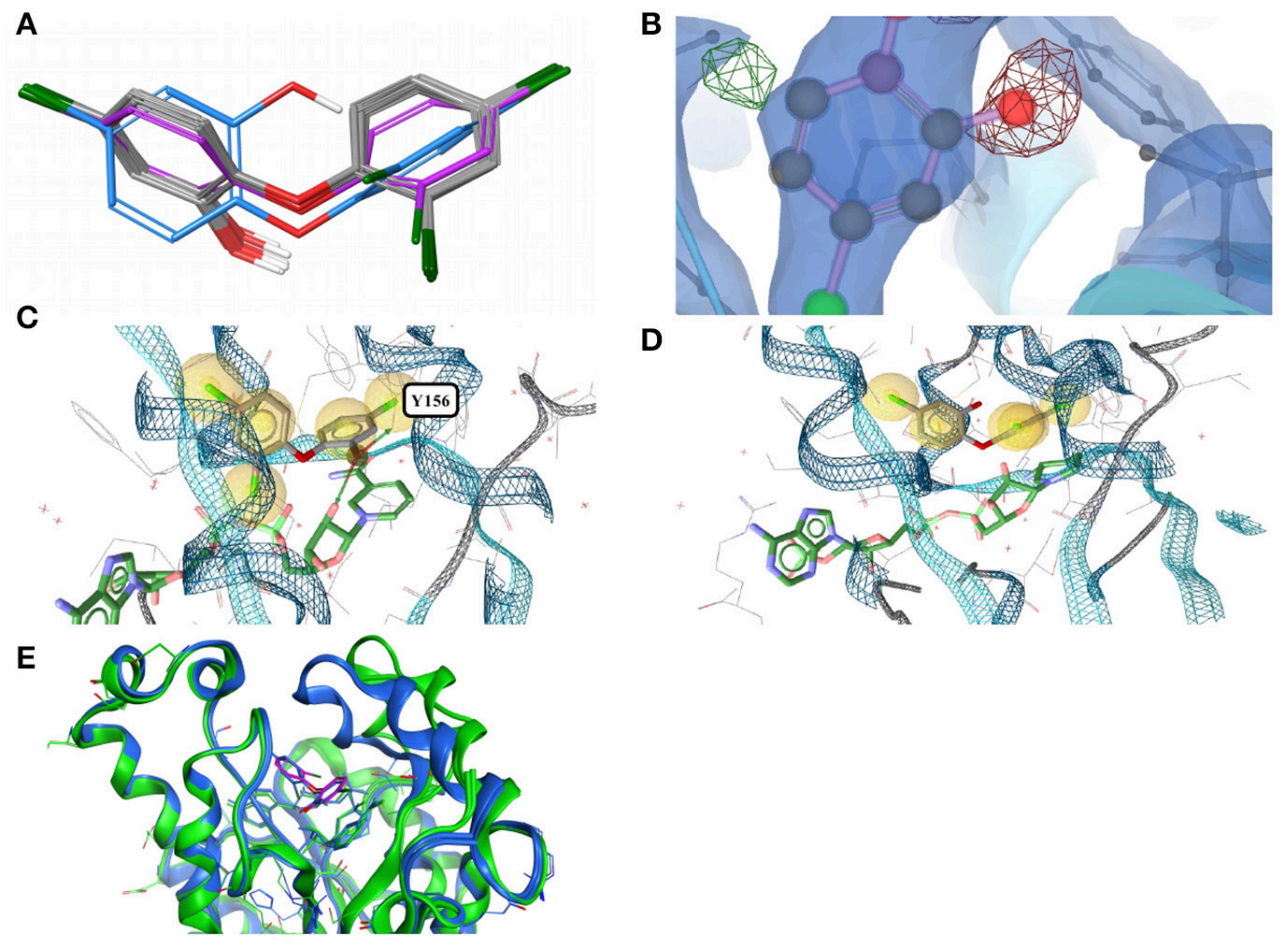
**(A)** Ligand-based alignment of eleven conformers of triclosan present in the Sperrylite Dataset bound to ENRs, including the drug-resistant G93V mutant of ENR I (3PJF; violet carbon atoms) and an uncommon conformation observed in *Staphylococcus aureus* ENR I (3GR6; blue carbon atoms). The latter is not part of the Sperrylite Dataset because of a lack of support of the structural model by the electron density, as shown in **(B)**, with the 2Fo-Fc, Fo-Fc(–ve) and Fo-Fc(+ve) sigma maps in blue, red and green, respectively. **(C)** Interaction of triclosan and NAD (green carbon atoms), including the characteristic hydrogen bond between both molecules in the binding pocket of *E. coli* ENR I (1QG6). **(D)** Triclosan and NADP (green) bound to *Staphylococcus aureus* ENR I (3GR6). In this structural model, the characteristic hydrogen bond is missing because of the unusual position of the hydroxyl group. However, this conformation of triclosan lacks support by the measured electron density. **(E)** A G93V mutation in ENR I (green protein backbone; ligand with violet carbon atoms) induces a conformational shift of the flexible α-helical turn located in the proximity of triclosan. The complex of the WT protein and triclosan (4M89) is shown with the protein backbone and ligand in blue.

In all 31 structures of triclosan contained in the PDB, the ligand is bound to an ENR. The conformers of triclosan observed among the 11 high-quality structures with ENR I and ENR III are very similar (median RMSD 0.1 Å; maximum pairwise RMSD < 0.6 Å; Figure 3A). These include the structures of *Plasmodium falciparum* ENR I (2O2Y) and *Bacillus subtilis* ENR III (3OID) which, despite a sequence identity of just 14% and a highly flexible binding site region (when in the unbound state), show almost identical structural features in the presence of triclosan (Kim et al., 2011).

In an X-ray structure of triclosan bound to *Staphylococcus aureus* ENR I (3GR6; not included in the Sperrylite Dataset because of low EDIA scores), the hydroxyl group of all four instances of triclosan is modeled in a different orientation (RMSD 1.4 Å measured to any of the other conformations present in the dataset). The EDIA score for the oxygen atom of the hydroxyl group of the four instances of this conformer is just 0.11–0.27, and visual inspection of the electron density map confirms a lack of support of this conformation (Figure 3B). The characteristic hydrogen bonds formed between the phenolic hydroxyl group of triclosan and Y156 as well as NAD(P) (Heath et al., 1999; Levy et al., 1999; Figure 3C) are also missing in this model (Figure 3D). All of these observations taken together indicate a likely error in this structural model.

The largest deviations between conformers of triclosan within the Sperrylite Dataset were observed for the complex with a triclosan-resistant G93V mutant (3PJF) of ENR I from *Escherichia coli*. These deviations are related to small conformational changes of a flexible α-helical turn in close proximity to the ligand (Figure 3E), resulting in the weakening of some edge-to-face aromatic interactions near the ligand (Singh et al., 2011). The high-level resistance of this mutant is not caused by a substantial loss in binding affinity of the drug but is a consequence of the inability of the G93V mutant to form the high affinity ENR-NAD+-triclosan ternary complex that inhibits the wild type (Heath et al., 1999).

#### Ubenimex, bestatin

Ubenimex, also known as bestatin (*BES*), is a competitive protease inhibitor under investigation for the treatment of acute myelocytic leukemia and lymphedema (Tian et al., 2017). The molecule inhibits aminopeptidases and has shown immunomodulatory and host-mediated antitumor activities (Urabe et al., 1993; Inoi et al., 1995; Sakuraya et al., 2000). It has been approved in Japan as an adjunct to chemotherapy agents against acute non-lymphocytic leukemia for decades and has been reported to inhibit the growth of malaria parasites (*Plasmodium falciparum*) *in vitro* (Nankya-Kitaka et al., 1998).

Twenty-eight structures of bestatin are listed in the PDB. All of the 11 high-quality structures are with bestatin bound to aminopeptidases. The ligand conformations observed in eight of these high-quality structures are very similar to each other (maximum pairwise RMSD = 0.8 Å), even though the proteins originate from three different bacteria (*E. coli, Pseudomonas putida* and *Vibrio proteolyticus*), the unicellular protozoan parasite *Plasmodium falciparum* and mouse, and their minimum pairwise sequence identity is only 3.3%.

**Figure 4:**
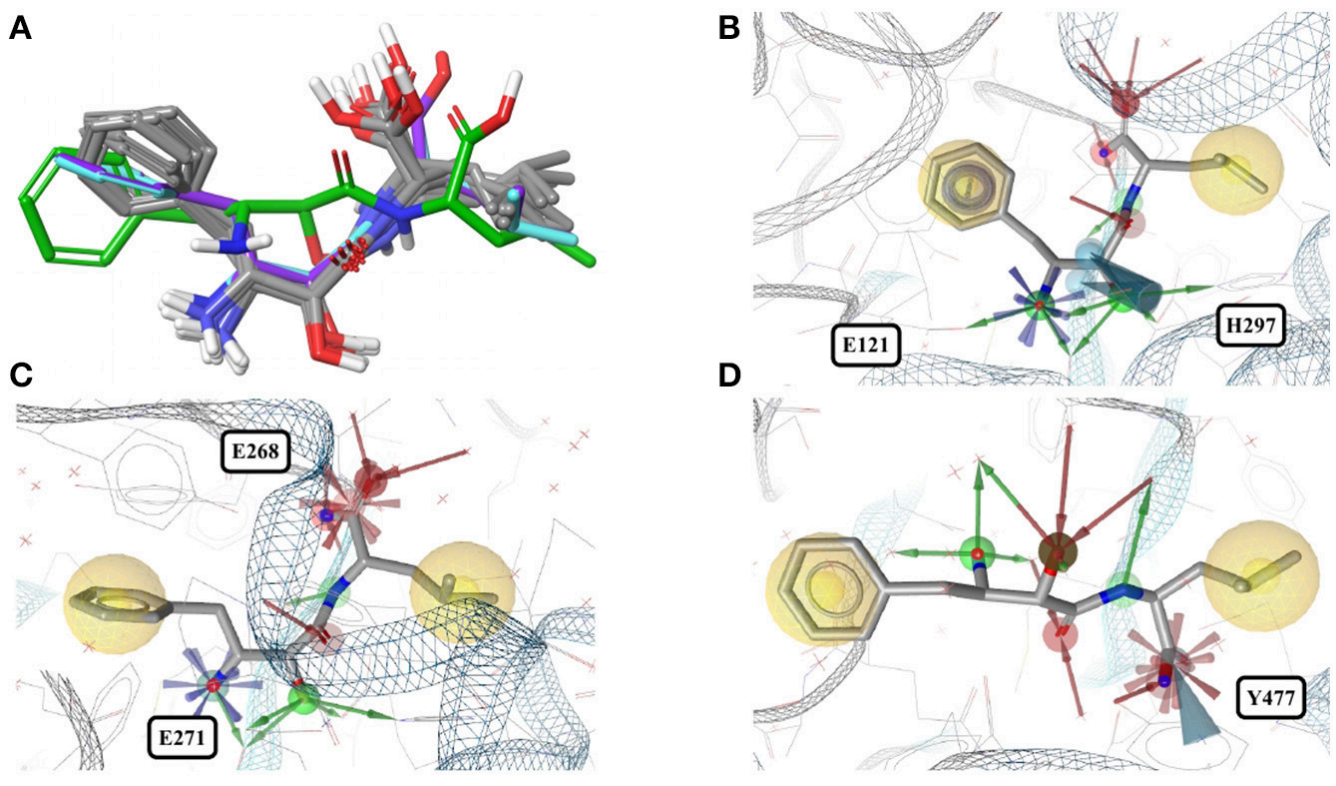
**(A)** Superposition of all eleven conformers of bestatin in the Sperrylite Dataset. The carbon atoms of the conformers in complex with human aminopeptidase N (4FYR) and human leukotriene A-4 hydrolase (3FUH and 3FTX) are indicated in green, violet and cyan, respectively. The carbon atoms of all other structures are shown in gray. **(B)** Typical conformer of bestatin bound to aminopeptidases N from *E. coli* (2HPT). **(C)** A conformation that differs slightly from the characteristic conformation, observed in complex with human leukotriene A-4 hydrolase (3FUH shown here). **(D)** Uncommon, extended conformation of bestatin observed in complex with the human aminopeptidase N (4FYR).

In contrast, the structure of bestatin bound to human aminopeptidase N (4FYR) shows an extended ligand conformation that has an RMSD of 2.0 Å to any of the ligand conformers observed for the bacterial proteins (Figure 4A). The conformations of the drug bound to human leukotriene A-4 hydrolase differ only slightly from and have similar binding modes to the characteristic conformation observed for aminopeptidases mentioned above (RMSD = 1.0 Å for both 3FUH and 3FTX; Figures 4B–D).

#### Biotin

**Figure 5:**
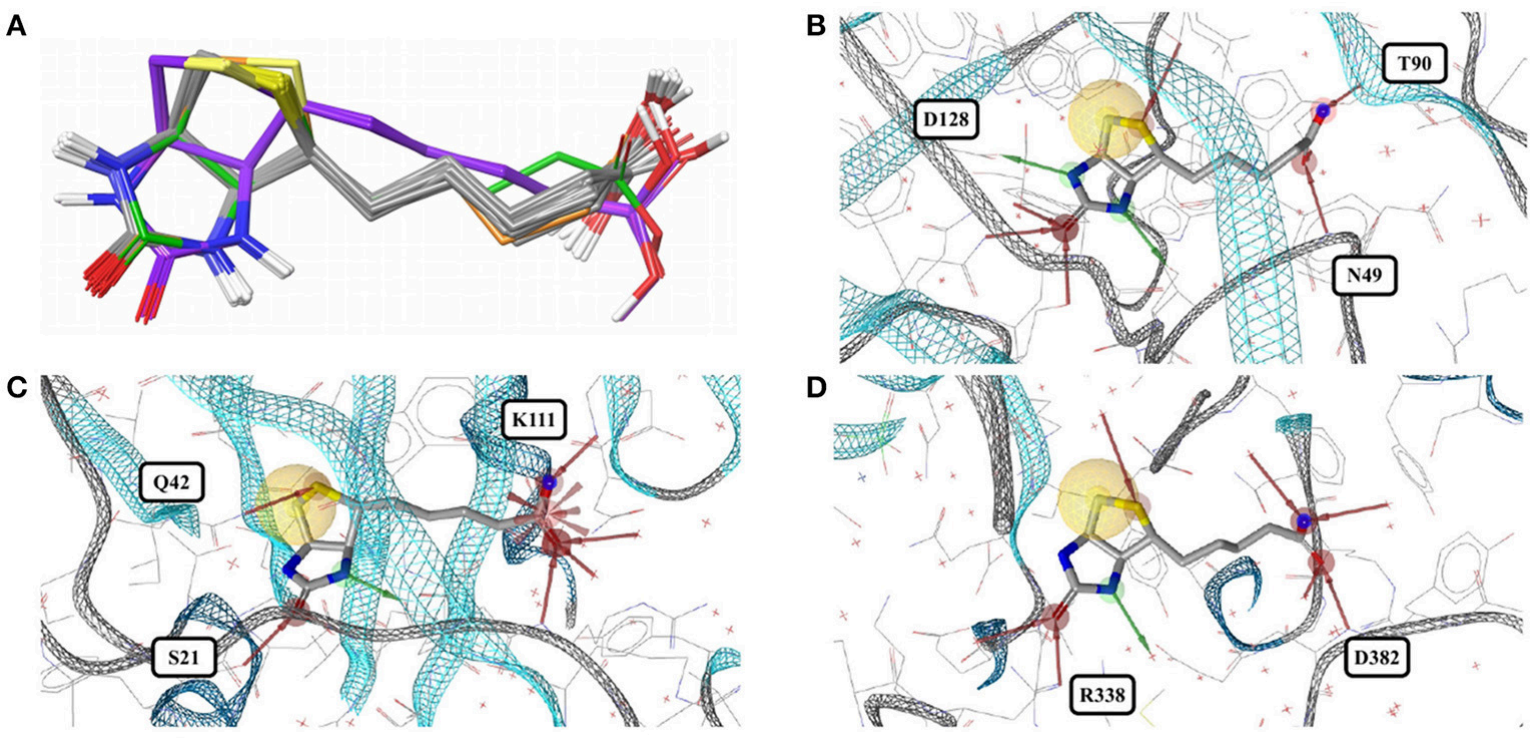
**(A)** Superposition of 43 structures of biotin (BTN) bound to core streptavidin (gray carbon atoms), *E. coli* biotin carboxylase (3G8C; green carbon atoms), biotin-protein ligase (1WPY; violet carbon atoms), and streptavidin-biotin at low pH (2IZJ; orange carbon atoms). The binding modes observed for biotin in complex with **(B)** core streptavidin from *Streptomyces avidinii* (3WYP), **(C)** biotin-protein ligase from *Pyrococcus horikoshii* (1WPY) and **(D)** *E. coli* biotin carboxylase (3G8C) are very similar.

Biotin (*BTN*, vitamin B_7_) is a water-soluble coenzyme for carboxylase enzymes and an approved drug for the treatment of dietary shortage or imbalance. There are 99 crystal structures including biotin listed in the PDB. The biotin conformers observed for the 43 high-quality structures can be assigned to three distinct groups, indicated by gray, green and violet carbon atoms in Figure 5A. Twenty-four of the 43 structures are complexes with core streptavidin from different bacteria (both wild type and mutants). Streptavidin homotetramers have a very high affinity for biotin, one of the strongest non-covalent interactions known (Kd ≈ 10^−14^ to 10^−16^ M) (Laitinen et al., 2006). The protein-ligand complex stands out by a high degree of shape complementarity and an extensive network of hydrogen bonds formed between both binding partners. One of the 24 structures of biotin bound to core streptavidin (4GD9) shows the impact of the cutting of a binding loop on the conformation of the bound ligand (Figure S4; Le Trong et al., 2013). Another structure (2IZJ) shows subtle structural changes of the streptavidin-biotin complex induced by a low pH that stabilizes intersubunit salt bridges (Figure 5A; orange carbon atoms; Katz, 1997).

Six crystal structures of avidin from chicken (wild type and mutants) and one of engineered avidin (2C4I) are also included in the dataset. Avidin is loosely related to streptavidin, with an equally high affinity to biotin and a very similar binding site (Figure S4). As expected, biotin binds to this protein in a conformation that is very similar to those predominantly observed for complexes with streptavidin.

Biotin-protein ligase (1WPY, 2EJ9, 2EJF, 2DTH, 2FYK, and 2ZGW) and biotin carboxylase (3G8C) share very low structural similarity with streptavidin and with each other. The conformations observed for biotin bound to biotin-protein ligase (Figure 5A; violet carbon atoms) are virtually identical among each other but differ by an RMSD of 1.1 Å from the predominant conformation observed in the Sperrylite Dataset. In particular, the angle of the alkyl chain leaving the ring system differs by around 103° from that observed for biotin bound to streptavidin. A third conformer of biotin is observed in complex with *E. coli* biotin carboxylase (3G8C; Figure 5A; green carbon atoms), with an RMSD of 0.9 Å measured against any of the streptavidin-bound conformers. Despite substantial structural differences observed among the various different biotin-binding proteins, the non-covalent interactions formed between biotin and the target protein are largely conserved (Figures 5B–D).

#### Sapropterin

Sapropterin (tetrahydrobiopterin, *H4B*) is an approved drug for the treatment of tetrahydrobiopterin deficiency. It is an essential cofactor for the synthesis of nitric oxide and the hydroxylation of phenylalanine, tyrosine and tryptophan. The PDB counts 472 complexes with sapropterin, 188 of which are of high quality.

Of the high-quality conformers of sapropterin, all but three are extremely similar to each other (median RMSD of less than 0.1 Å; Figure S5A). All of these highly similar sapropterin conformers are bound to nitric oxide synthase, from five different species (human, rat, mouse, cattle and *Bacillus subtilis*). The exceptions are the conformers bound to human phenylalanine hydroxylase (1MMK, 1MMT and 1J8U), and differ by an RMSD of 0.7 Å from the conformer in human nitric oxide synthase (4D1N, Figure S5B). The sequence identity between human phenylalanine hydroxylase and human nitric oxide synthase is less than 15%. The slightly different conformer bound to phenylalanine hydroxylase is stabilized by hydrophobic interactions (Figure S5C).

#### Cholic acid

Cholic acid (*CHD*) is one of the major bile acids produced from cholesterol in the liver. It is approved for the treatment of bile acid synthesis disorders and as an adjunctive treatment of peroxisomal disorders.

Thirteen of the 74 available crystal structures that include cholic acid are of high quality. Twelve thereof are from eukaryotic proteins, including alcohol dehydrogenase, ferrochelatase, cytochrome c oxidase and bile acid-binding proteins; one structure is of choloylglycine hydrolase from *Clostridium perfringens* (2RLC).

Some pockets of cholic acid-binding proteins can accommodate more than a single cholic acid molecule, as observed e.g., in structures of the chicken liver basic fatty acid-binding protein (1TW4) and the zebrafish liver bile acid-binding proteins (2QO5).

**Figure 6:**
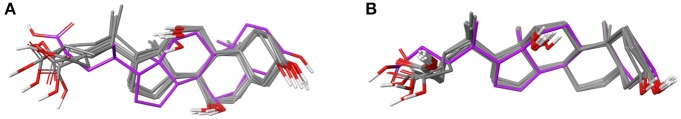
**(A)** Ligand-based alignment of 13 structures of cholic acid bound to different eukaryotic proteins and choloylglycine hydrolase from *Clostridium perfringens* (2RLC; gray carbon atoms) and human mitochondrial ferrochelatase (3W1W; violet carbon atoms). **(B)** Ligand-based alignment of 16 structures of deoxycholic acid bound to structurally distinct proteins, including salmonella invasion protein D (3O01; violet carbon atoms).

Given the rigid scaffold of steroids it is not surprising that, despite in part low sequence identity between the cholic acid-binding proteins, the observed ligand conformations (i.e., those bound to the deepest part of their respective binding pocket) are highly similar (median RMSD = 0.6 Å; Figure 6A). The maximum pairwise RMSD of 1.6 Å was measured between the conformation of cholic acid in the crystal structure of the G55R mutant of zebrafish liver bile acid-binding protein (2QO6) and in human mitochondrial ferrochelatase (3W1W).

#### Deoxycholic acid

Deoxycholic acid (*DXC*), a metabolic byproduct of intestinal bacteria, is a steroid acid commonly found in the bile of mammals (Ridlon et al., 2016). Deoxycholic acid is a detergent that disturbs the integrity of biological membranes and is used to isolate membrane-associated proteins. Deoxycholic acid is approved for submental fat reduction, as a safer and less invasive alternative to surgical procedures for the treatment of lipomas (Duncan and Rotunda, 2011) and for improvements of aesthetic appearance.

Of the 29 entries deposited in the PDB, 18 are of high quality. Eleven of those structures are deoxycholic acid bound to cathepsin A and have a maximum pairwise RMSD of just 0.1 Å. Because of the rigid ligand core, deoxycholic acid also binds to structurally distinct proteins in very similar conformations (Figure 6B). Examples from the Sperrylite Dataset include two structures of *Betula pendula* Bet v1 (a major pollen allergen; 4A81 and 4A84), a structure of subunits I and II of cytochrome c oxidase (3DTU) from *Rhodobacter sphaeroides*, a structure of choloylglycine hydrolase from *Clostridium perfringens* (2BJF), a structure of the multidrug transporter MdfA (4ZP0) from *E. coli*, and even a conformer of deoxycholic acid bound to the interface of a dimer of the cell invasion protein SipD from *Salmonella enterica* (3O01; Chatterjee et al., 2011) The maximum pairwise RMSD (0.9 Å) was measured for the ligand conformers bound to a K9E mutant of cathepsin A (4HAJ) and salmonella invasion protein D (3O01), indicated by violet carbon atoms in Figure 6B.

### Cofactors and cofactor analogs

**Figure 7:**
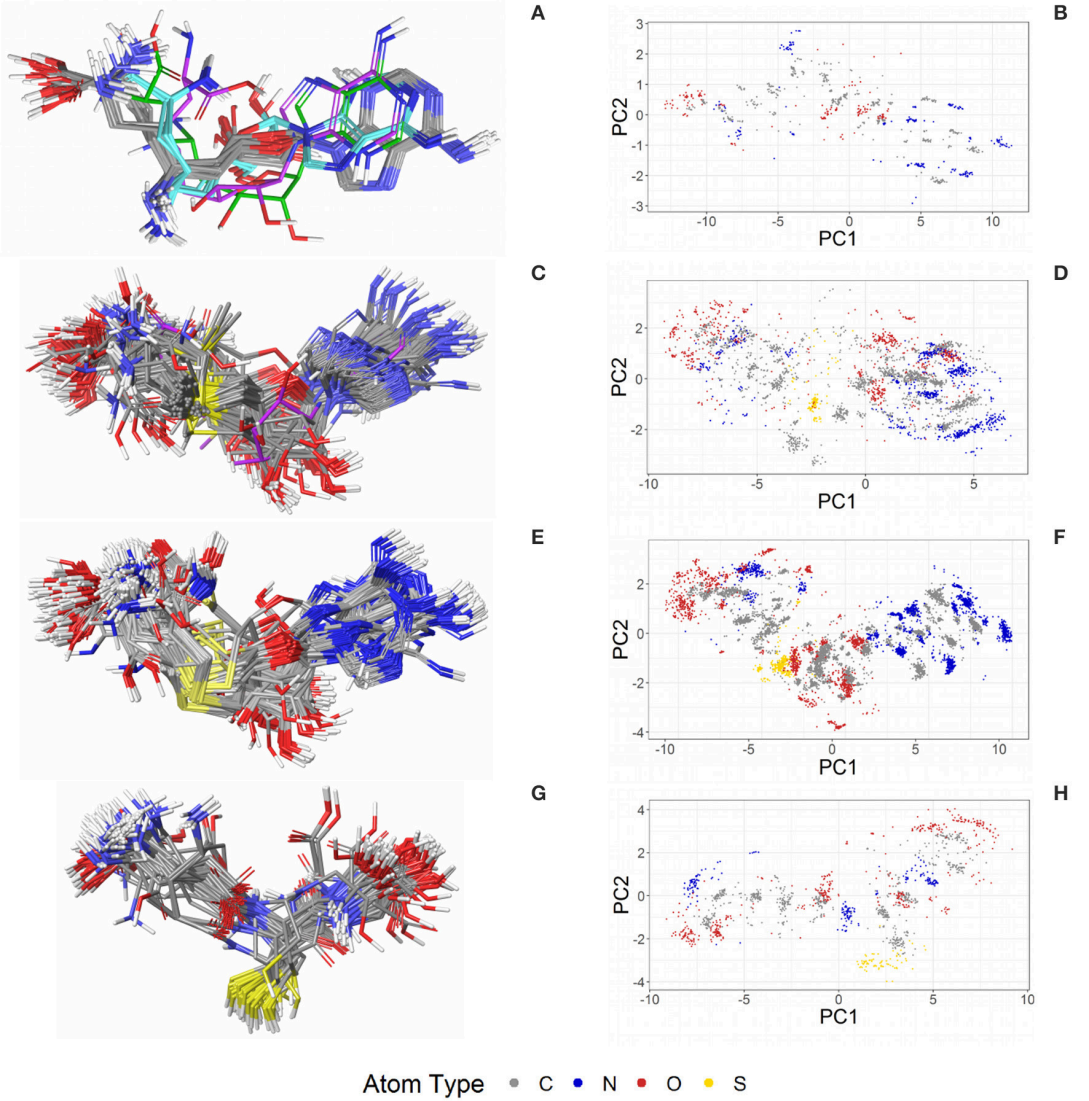
Ligand-based alignment (left) and PCA-derived score plots (right) of **(A,B)** 30 structures of sinefungin bound to different methyltransferases (gray carbon atoms; these and all further color definitions in this caption are referring to the left panels only), ribosomal RNA small subunit methyltransferase NEP1 (3BBH; violet carbon atoms), tRNA (guanine-N(1)-)-methyltransferase (4YVH; green carbon atoms), SMYDs and SET7 lysine methyltransferase (3CBP, 3PDN, 3N71, 3QWW and 3RU0; cyan carbon atoms); **(C,D)** 123 structures of SAM bound to different methyltransferases (gray carbon atoms), tRNA(m1G37)methyltransferase (1UAK; violet carbon atoms) and yeast ribosome synthesis factor Emg1 (2V3K; green carbon atoms); **(E,F)** 311 structures of SAH and **(G,H)** 74 conformers of glutathione (GSH).

**Figure 8:**
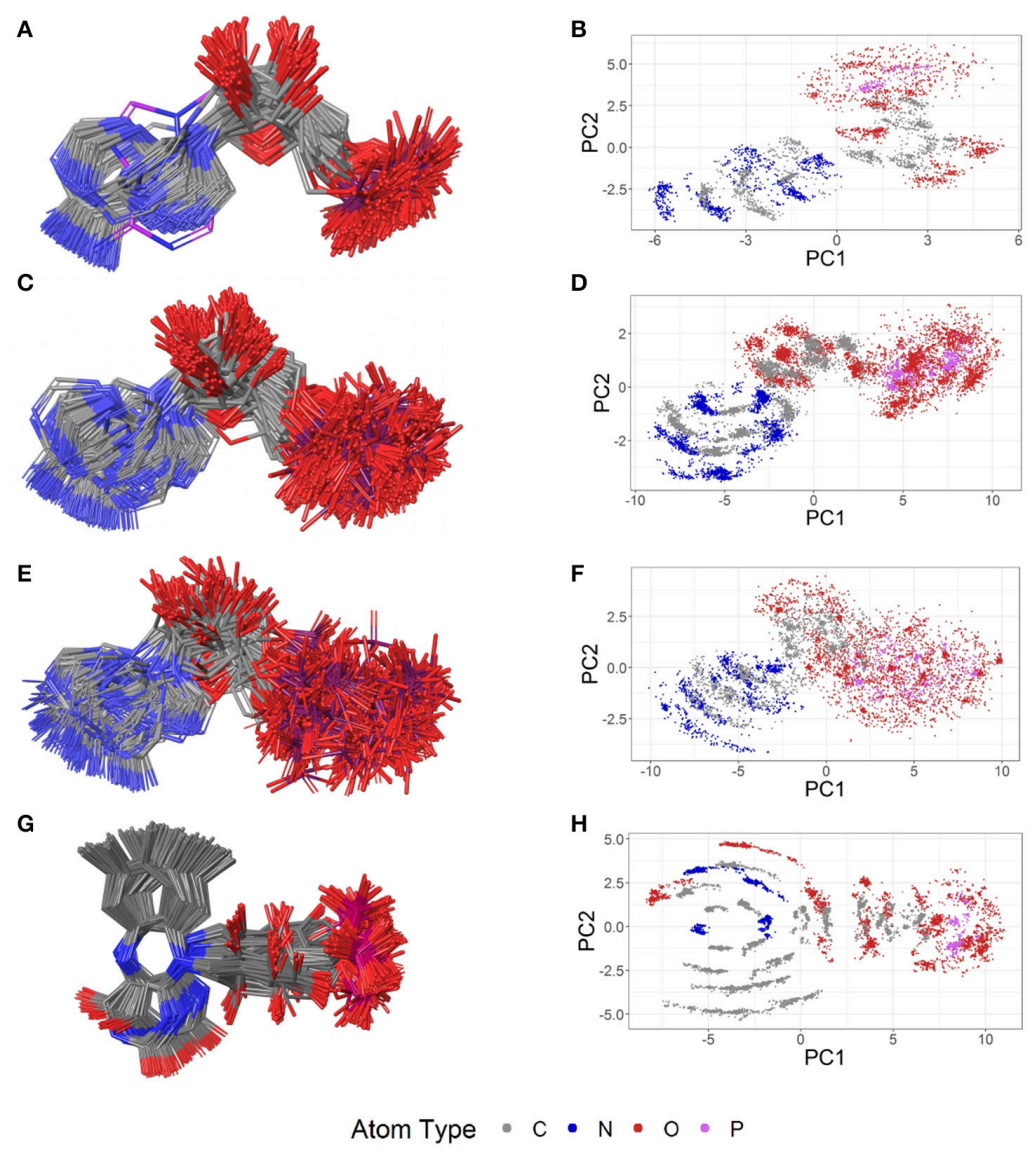
Ligand-based alignment (left) and PCA-derived score plots (right) of **(A,B)** 171 conformers of AMP (conformer bound to adenylate kinase-related protein from *Sulfolobus solfataricus* in **(A)** with violet carbon atoms; 3LW7), **(C,D)** 462 conformers of ADP, **(E,F)** 218 conformers of ATP, and **(G,H)** 367 conformers of FMN.

**Figure 9:**
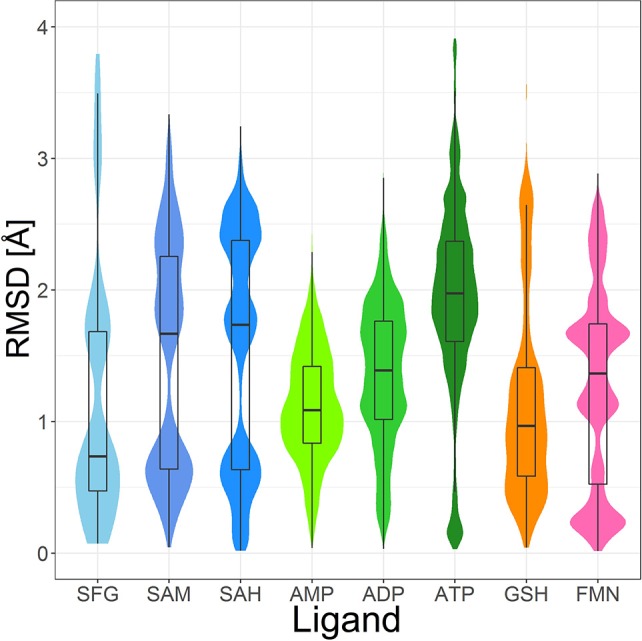
Violin plot including box plots of the RMSD distributions of high-quality, protein-bound conformations of sinefungin (SFG), SAM, SAH, AMP, ADP, ATP, GSH and FMN. The width of each violin plot for a certain RMSD value indicates how often the specific value occurs in the pairwise comparison of all conformers.

The most abundant small molecules in the Sperrylite Dataset are cofactors and their analogs. The cofactors represented by at least 10 high-quality structures can roughly be grouped into three categories: sinefungin and its analogs (S-adenosylmethionine, SAM, and S-adenosylhomocysteine, SAH; Figure 7), adenosine phosphates (AMP, ADP, ATP; Figure 8), and three cofactors without analogs listed in the dataset (glutathione, flavin mononucleotide and sapropterin). The RMSD distributions (all-against-all comparisons) for the most relevant cofactors are reported in Figure 9.

#### Sinefungin and analogs

##### Sinefungin

Sinefungin (*SFG*), an analog of the cofactor substrate SAM, inhibits a wide range of methyltransferases, thereby interfering with DNA synthesis (Pugh et al., 1978). It is an antifungal antibiotic and also a known effective inhibitor of the transformation of chick embryo fibroblasts by the cancer-causing *Rous sarcoma* virus (Vedel et al., 1978).

**Figure 10:**
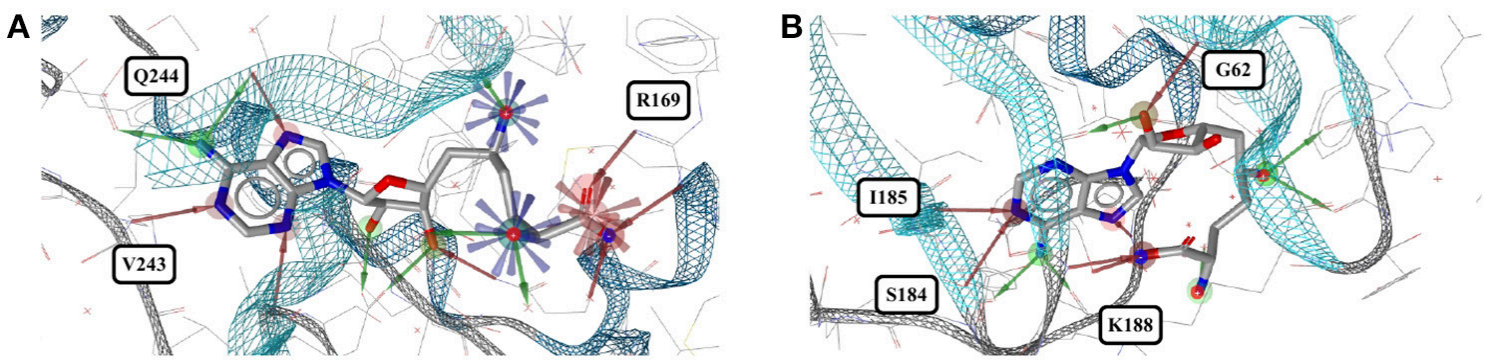
**(A)** A typical conformer of sinefungin bound to human histone-arginine methyltransferase CARM1 (2Y1W) and **(B)** the coiled conformer in the ribosomal RNA small subunit methyltransferase NEP1 from *Methanocaldococcus jannaschii* (3BBH).

The PDB lists 70 structures of sinefungin, all of them bound to methyltransferases. Thirty of these structures are of high quality. The observed conformers of sinefungin can be classified into three groups by an all-against-all comparison of their RMSDs (Figure 9). The largest group (Figure 7A; gray carbon atoms) includes 23 highly similar conformers (a representative example is given in Figure 10A) with a median RMSD of 0.5 Å, even though some of the proteins that these sinefungin molecules are bound to share low sequence identity (e.g., 30% for murine protein arginine N-methyltransferase 6 and the ribosomal protein L11 methyltransferase of *Thermus thermophilus*).

The second largest group consists of sinefungin conformers bound to the murine SET and MYND domains (SMYD) 1 (3N71) and 2 (3QWW), the human SMYD 3 (3PDN, 3RU0) and the SET7 lysine methyltransferase (3CBP), with RMSDs between 1.7 and 1.8 Å measured against the conformations representing the largest group (Figure 7A; cyan carbon atoms). Murine SMYD 1 (3N71) and human SET7 lysine methyltransferase (3CBP) have less than 15% sequence identity but bind sinefungin in very similar conformations (RMSD 0.3 Å).

Distinct conformations of sinefungin are observed for a complex with *Haemophilus influenzae* tRNA (guanine-N(1)-)methyltransferase (4YVH; Figure 7A; green atoms) and a complex with the ribosomal RNA small subunit methyltransferase NEP1 (3BBH, Figure 7A; violet carbon atoms, and Figure 10B) from *Methanocaldococcus jannaschii*, with RMSDs measured to the most abundantly observed conformation of 3.1 and 3.6 Å, respectively. In both cases the ligand conformation is stabilized by a hydrogen bond formed between the ligand's carboxyl group and the protein backbone.

##### S-adenosylmethionine

SAM (*SAM*) is a cofactor that functions as a methyl donor in methyltransferases. It is essential for the methylation of proteins, DNA, lipids and small molecules. The bulk of SAM is generated in the liver, but all mammalian cells use it as an intermediate in the methionine-homocysteine cycle (Mato et al., 2013). SAM is also involved in the synthesis of many other endogenous metabolites. It has wide-ranging anti-inflammatory activity (Pfalzer et al., 2014) and, since its synthesis is depressed in chronic liver diseases, there has been considerable interest in its therapeutic use (Anstee and Day, 2012; Guo et al., 2015). S-adenosylmethionine is used as a drug for the treatment of depression, liver disorders, fibromyalgia, and osteoarthritis.

Four hundred ten structures listed in the PDB contain SAM. For example, almost all crystal structures of flavivirus methyltransferases contain SAM (because the molecule co-purifies with the enzymes (Noble et al., 2014). There are 119 high-quality SAM-containing structures present in the Sperrylite Dataset. Many of these conformers are similar, with an overall median RMSD of 0.6 Å (Figures 7C,D). Even conformers bound to proteins sharing a low sequence identity (e.g., 19% in the case of *Aeropyrum pernix* fibrillarin, 4DF3, and human NSUN5, 2B9E), have RMSDs of just 0.5 Å. The all-against-all RMSD comparison shows a partitioning into three groups that are mainly determined by the torsion angles between the adenine and the ribose and to the torsion angles including the sulfonium linkage (Figure 7). The highest RMSD measured between any pair of SAM conformers is 3.3 Å, which was measured for the ligand in complex with *Haemophilus influenzae* tRNA(m1G37)methyltransferase (1UAK; Figure 7C; violet carbon atoms) and with SAM methyltransferase from *Ruegeria pomeroyi* (3IHT).

##### S-adenosyl-L-homocysteine

The strong product inhibitor SAH (*SAH*) is released in all SAM-dependent methyltransferase reactions (Tehlivets et al., 2013). The ratio of SAM to SAH controls the activity of methyltransferase enzymes (“methylation ratio”; Schatz et al., 1977).

The PDB lists 784 structures including SAH, of which an unusually high proportion (40%; 311 structures) is of high quality (Figure 7). These represent a highly diverse set of proteins from all three domains of organisms in nature. Most of the structures are of human (73 structures) and *Pyrococcus horikoshii* (72 structures) proteins.

Many of the SAH conformations are highly similar, with an overall median RMSD of 0.6 Å. The all-against-all RMSD comparison shows three groups of conformations and an overall spread very similar to that observed for SAM (Figure 9). As shown in Figure 7, the conformations observed for SAM and SAH are similar. Also, all conformations of sinefungin are closely represented by at least one conformation of SAM and SAH.

The largest difference observed among the SAH conformations was measured between a coiled conformer bound to *Haemophilus influenzae* tRNA (Guanine-N(1)-)-methyltransferase (1UAL) and a mostly stretched conformer bound to *E. coli* ribosomal RNA large subunit methyltransferase L (3V97) with an RMSD of 3.2 Å.

#### Glutathione

**Figure 11:**
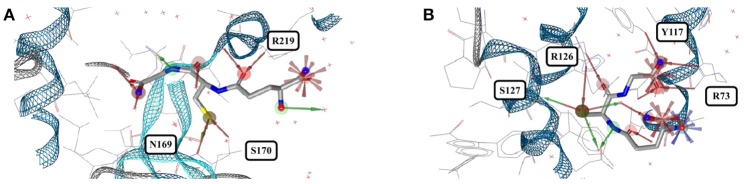
**(A)** The most stretched conformer of glutathione bound to an ABC transporter from *Chromobacterium violaceum* (4PYR) and **(B)** an unusually coiled conformer of glutathione bound to human mPGES-1 (4YL1).

The tripeptide glutathione (GSH; *GSH*) is a cofactor of various different enzymes and a defensive reagent against toxic xenobiotics. Of the 360 entries with glutathione listed in the PDB, 74 structures are of high quality. These high-quality structures cover glutathione bound to 10 different proteins (Figures 7G,H). Most of the GSH conformers have a pairwise RMSD between 0.6 and 1.6 Å (Figure 9). The two most distinct conformers of glutathione observed in the Sperrylite Dataset are an unusually stretched conformer bound to a putative branched-chain amino acid ABC transporter from *Chromobacterium violaceum* (4PYR, Figure 11A) and an extremely coiled conformer bound to human mPGES-1 (4YL1, Figure 11B), with an RMSD of 3.6 Å. Nevertheless, their interaction patterns show similarities. Glutathione transferases are represented by 46 high-quality structures. These are mostly similar and have a median RMSD of less than 0.5 Å (Figures 7G,H).

#### Adenosine phosphates

ATP functions as the most important molecule for intracellular storage and transport of chemical energy. It has many crucial roles in metabolism and is also a neurotransmitter. During metabolic processes, ATP is converted into adenosine diphosphate (ADP) and, subsequently, adenosine monophosphate (AMP), thereby releasing the stored energy.

##### Adenosine monophosphate

**Figure 12:**
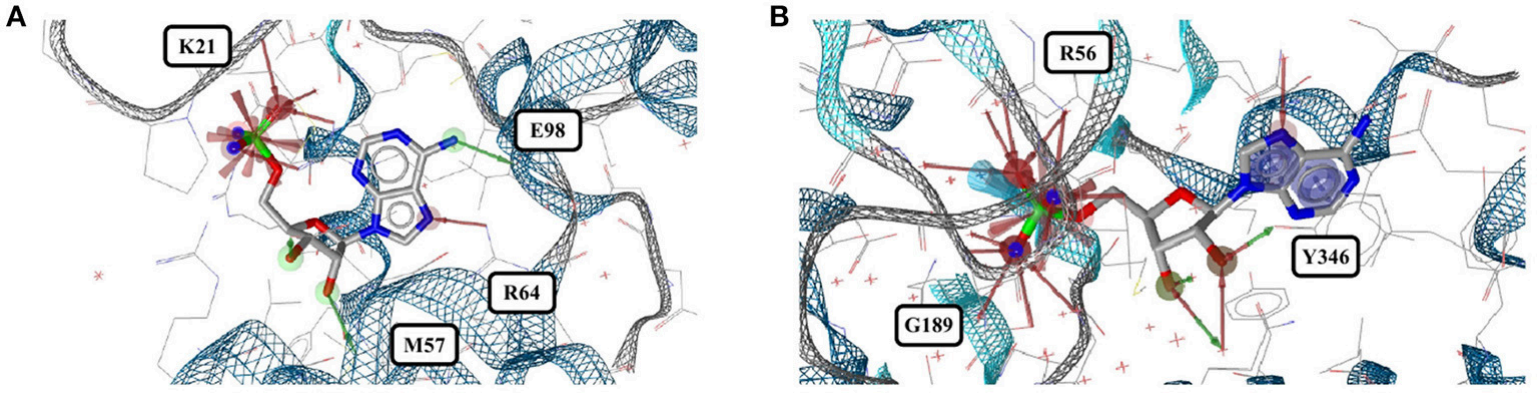
**(A)** Unusually coiled conformer of AMP bound to adenylate kinase-related protein of *Sulfolobus solfataricus* (3LW7) and **(B)** the most stretched conformer in *Legionella pneumophila* NTPDase1 (4BRN).

Out of the 575 complexes with AMP (*AMP*) found in the PDB, 171 conformers are of high quality. AMP has four rotatable bonds and the median RMSD measured between all high-quality conformers is 0.8 Å. The all-against-all comparison of AMP conformers results in a wide spread of the RMSD values (Figure 9). The flexibility of the molecule is mostly limited to the phosphate group (Figures 8A,B). The maximum RMSD of 2.5 Å was measured between an extremely coiled conformer bound to an adenylate kinase-related protein from *Sulfolobus solfataricus* (3LW7; Figure 8A, violet carbon atoms; Figure 12A) and the stretched conformer bound to NTPDase1 from *Legionella pneumophila* (4BRN; Figure 12B).

##### Adenosine diphosphate

**Figure 13:**
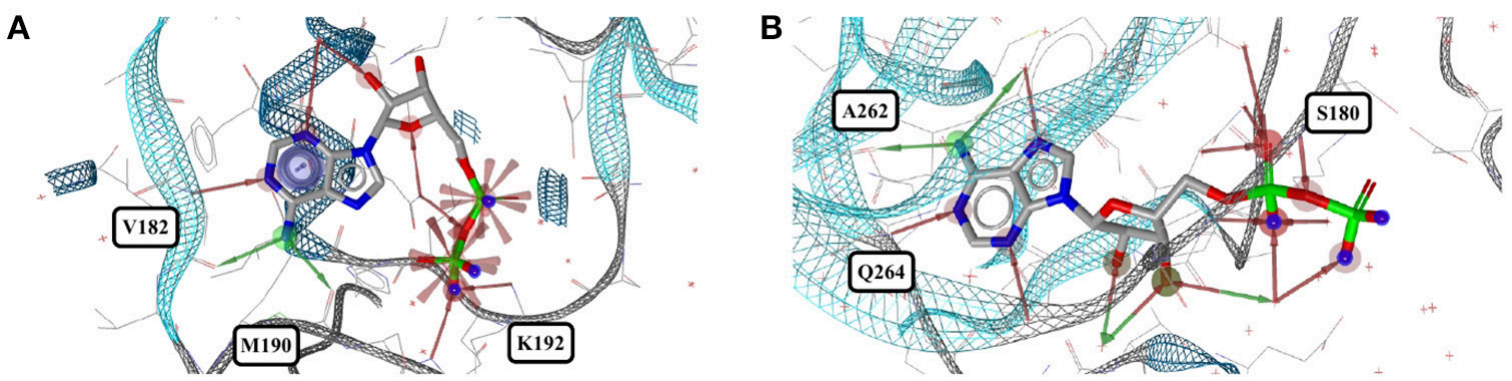
The most distinct conformers of ADP in the Sperrylite Dataset are the coiled conformer from **(A)** tryptophanyl-tRNA synthetase from *Campylobacter jejuni* (3TZL; sodium ion in light blue) and **(B)** the stretched conformer from a Stt7 homolog from *Micromonas algae* (4IX6).

Out of the 1,810 entries including ADP (*ADP*) in the PDB, 462 conformers are of high quality. Despite an additional phosphate group and a total of six rotatable bonds, the conformational space covered by ADP is very similar to that covered by AMP (Figures 8C,D). This similarity is reflected in the median RMSD of 0.9 Å between the conformers of ADP and a similar overall spread in the all-against-all comparison (Figure 9). The two most different ADP conformers in the Sperrylite Dataset are those bound to tryptophanyl-tRNA synthetase from *Campylobacter jejuni* (3TZL; Figure 13A) and an Stt7 homolog from *Micromonas algae* (4IX6; Figure 13B), with an RMSD of 2.9 Å.

##### Adenosine triphosphate

Only 218 conformers out of the 1,079 structures of the PDB containing ATP (*ATP*) were of high quality. In all structures of ATP included in the Sperrylite Dataset, the N-glycosidic bond is found in an anti-orientation. With its eight rotatable bonds ATP is more flexible than the previously discussed adenosine phosphates. This results in a median RMSD of 1.6 Å among the ATP structures of the Sperrylite dataset (as compared to a median RMSD of 0.9 Å measured for ADP) and a distinct spread of the RMSD values in the all-against-all comparison (Figure 9). The maximum pairwise RMSD was 3.9 Å, measured between ATP conformers from human lysyl-tRNA synthetase (3BJU) and *Drosophila melanogaster* Wiskott-Aldrich syndrome protein homology 2 (3MN6).

ATP is observed in an extended conformation in most structures (Figures 8G,H), which is in agreement with earlier studies (Moodie and Thornton, 1993; Stockwell and Thornton, 2006; Bojovschi et al., 2012; Stegemann and Klebe, 2012). As reported also by Stockwell and Thornton (Stockwell and Thornton, 2006), some conformers are bent to an extent that the terminal phosphate atoms are almost in van der Waals contact with the adenine ring. Examples of ATP in bent conformations include complexes with the aspartyl-tRNA synthetase from *Pyrococcus kodakaraensis* (1B8A; Figure S6) and the ribonucleotide reductase protein R1 from *E. coli* (3R1R).

#### Flavin mononucleotide

Flavin mononucleotide (FMN; *FMN*) is the prosthetic group of various oxidoreductases (including NADH dehydrogenase), as well as a cofactor in biological blue-light photoreceptors (Froehlich et al., 2002; Schwerdtfeger and Linden, 2003). Blue-light receptors in plants (phototropins), for example, employ flavin mononucleotide as the chromophore for their light sensing function (He, 2002).

Its frequent occurrence as a prosthetic group and a cofactor result in flavin mononucleotide's presence in 919 structures deposited in the PDB, among which 367 conformers of FMN are of high quality. Despite having seven rotatable bonds, most structures show extended, similar conformations (Figures 8G,H), with a median RMSD of 0.9 Å. The all-against-all comparison reveals four groups of conformers, with peaks observed in the RMSD distribution around 0.3, 1.2, 1.7, and 2.4 Å (Figure 9). These peaks correspond to an accumulation of conformers with similar torsion angles of the side chain. The maximum RMSD of 2.9 Å was observed between the conformation of FMN in *E. coli* pyridoxine 5′-phosphate oxidase (1JNW) and in human glycolate oxidase (2RDU), with the sidechain bent into opposing directions.

## Conclusions

The Sperrylite Dataset presented in this work is a complete subset of high-quality conformations of protein-bound ligands extracted from the PDB. This dataset resulted from a multi-step data processing and filtering procedure that, most importantly, also includes an automated approach for the evaluation of the support of individual atom positions by the electron density. The Sperrylite Dataset consists of a total of 10,936 high-quality structures of 4,548 unique ligands. Ninety-one of those ligands are each represented by a minimum of ten structures, and among these only a (very) weak correlation was observed between the number of rotatable bonds of a molecule and its overall variability (measured as the minimum median RMSD; *R*^2^ = 0.126). Sixty-nine out of the 91 ligands had at least two distinct conformations (defined as RMSD above 1 Å).

A representative subset of 17 approved drugs and cofactors was analyzed in detail to determine the conformational variability of protein-bound conformations of small molecules. For all of the analyzed small-molecule drugs and some of the cofactors, a clear trend for the formation of few clusters of highly similar conformers was observed. Similar conformers were observed for proteins with similar binding sites, mostly independent of the overall protein sequence identity (which is in agreement with the findings of, e.g., Sturm et al., 2012). A particularly interesting example is imatinib, which was found to adopt highly similar conformations when binding to different tyrosine kinases (even to those sharing low overall sequence identity) but to adopt a distinct conformation upon binding to quinone reductase 2. For cofactors, a clear trend for extended conformations was observed, which is in agreement with previous works (Moodie and Thornton, 1993; Stockwell and Thornton, 2006; Bojovschi et al., 2012; Stegemann and Klebe, 2012). A few cases of strongly coiled conformers of cofactors were also observed. This result is well in line with earlier reports (Stockwell and Thornton, 2006).

It is clear that the currently available structural data on protein-bound ligands is still too limited to allow us to gain a full understanding of the bioactive space of small molecules. However, for several cofactors a large number of conformers observed in complex with dozens of proteins are available to date and provide valuable insight into the bioactive conformational space and the prevalence of bioactive conformations of small molecules. With an automated workflow for the extraction of high-quality ligand structures from the PDB in place, it is expected that the ever increasing amount of data will allow a more detailed understanding of, e.g., conformational preferences, ligand promiscuity, or the relationship between the bioactive conformational space of small molecules and the structural diversity of binding pockets.

## Data availability

The dataset generated for this study can be found at: http://www.zbh.uni-hamburg.de/sperrylite_dataset.

## Author contributions

JK and N-OF: conceived the work; N-OF and MS: conducted the computational studies. All authors contributed to the interpretation of the data and the writing of the manuscript. All authors have given approval to the final version of the paper.

### Conflict of interest statement

The authors declare that the research was conducted in the absence of any commercial or financial relationships that could be construed as a potential conflict of interest.
