# Supplementary material for: How Diverse Are the Protein-Bound Conformations of Small-Molecule Drugs and Cofactors?
Source: Front Chem. 2018 Mar 27;6:68. doi: 10.3389/fchem.2018.00068 (PMC5880911; doi:10.3389/fchem.2018.00068)
Supplement: Supplementary file 1 [file DataSheet1.PDF]

Supplementary Material

How Diverse are the Protein-Bound Conformations of Small-Molecule Drugs and Cofactors?

Nils-Ole Friedrich, Méliné Simsir, Johannes Kirchmair*

*** Correspondence:** J. Kirchmair
E-mail: kirchmair@zbh.uni-hamburg.de
Tel.: +49 (0)40 42838 7303

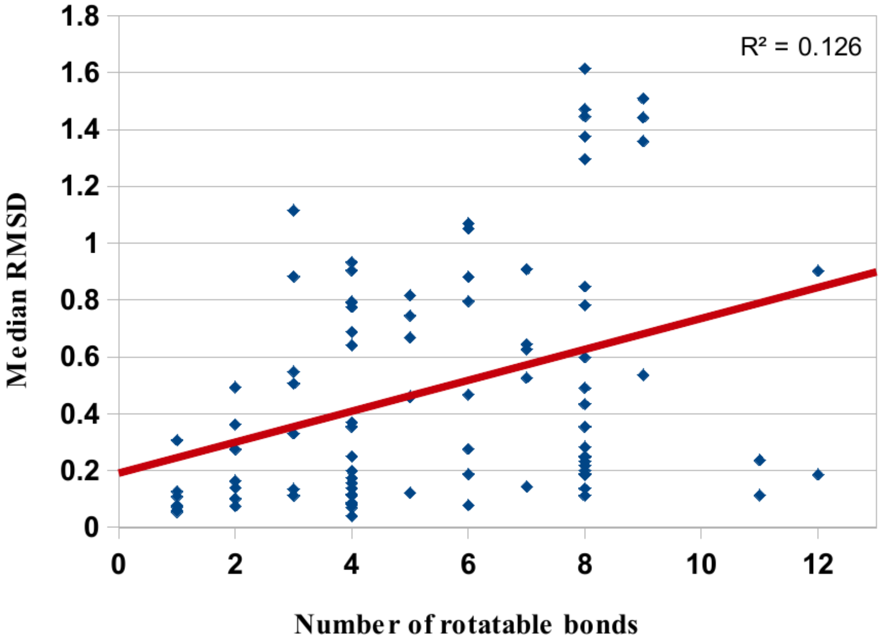

Figure S1. Minimum median RMSD values plotted against the number of rotatable bonds.

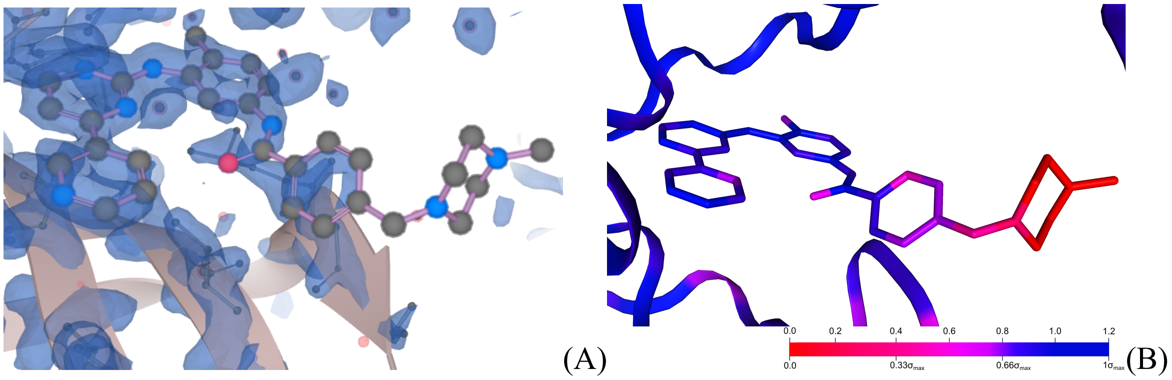

Figure S2. (A) Weak electron density support for parts of imatinib bound to human SYK modeled in an uncommon orientation (1XBB; $EDIA_m$ = 0.21). (B) The EDIA score is indicated by a color gradient, ranging from dark red (no or poor electron density support) via magenta to blue (good electron density support). For single atoms, EDIA values above 0.8 mark well-supported atoms, values in the range of 0.4 to 0.8 atoms with medium support and values below 0.4 poorly-supported atoms. For the EDIAm, the developers of the method concluded that only structures with a score higher than 0.8 should be considered as well supported by the electron density.

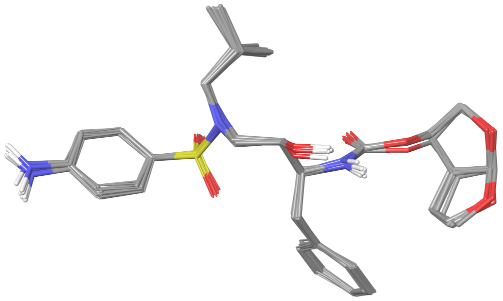

Figure S3. Ligand-based alignment of all 14 conformers of darunavir present in the Sperrylite Dataset.

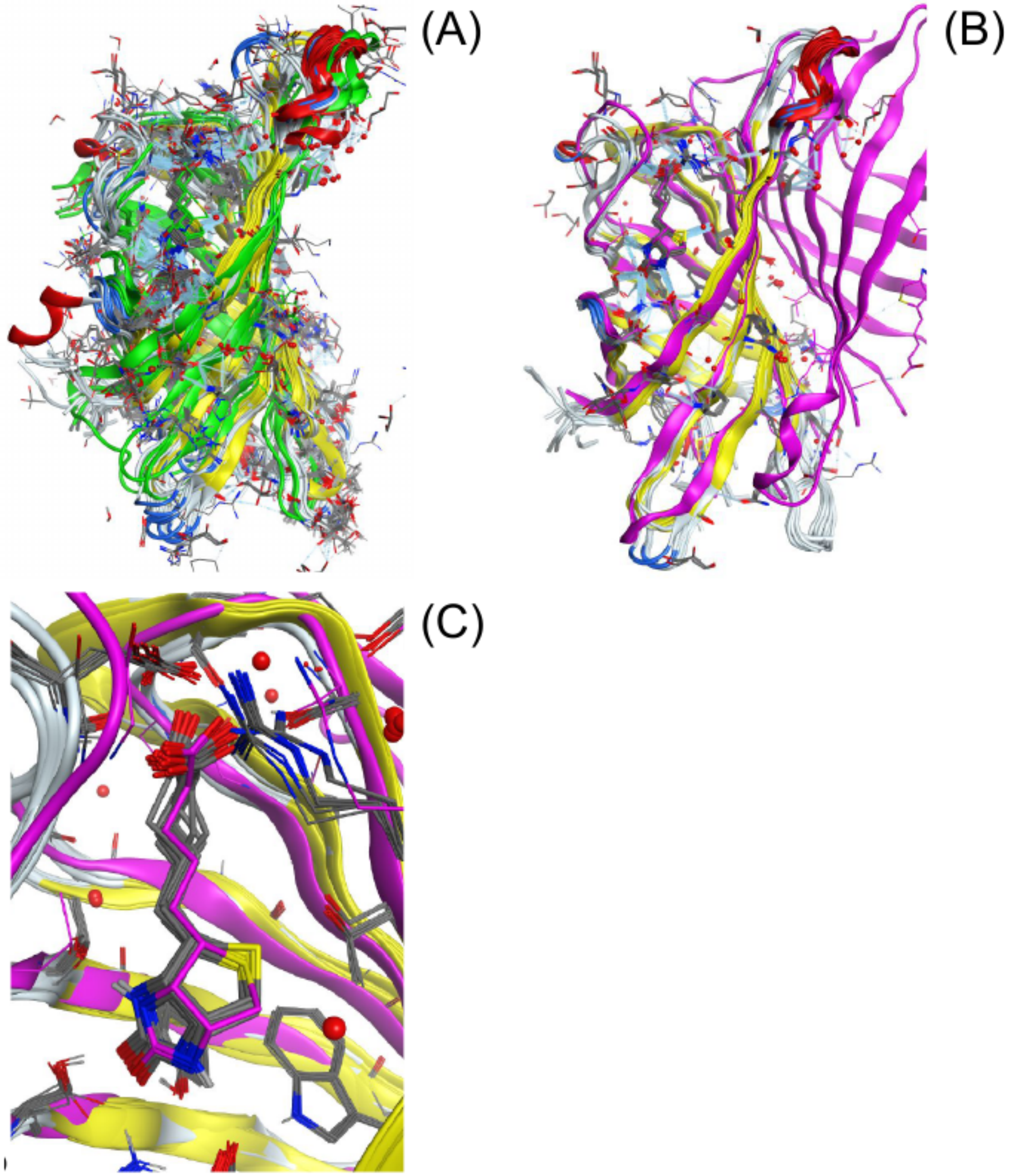

Figure S4. (A) Superposition of all 34 high-quality core streptavidin structures (including tetramers) bound to biotin. The four high-quality structures of the streptavidin N49/G48 mutant from *Streptomyces avidinii* (4GD9) in green show the structural consequences of cutting a binding loop. (B) Ten high-quality core streptavidin structures superposed with the loosely-related crystal structure of engineered dual chain avidin (2C4I), in violet, (C) with very similar binding modes. In dual chain avidin, two circularly permuted chicken avidin monomers are fused into one polypeptide chain.

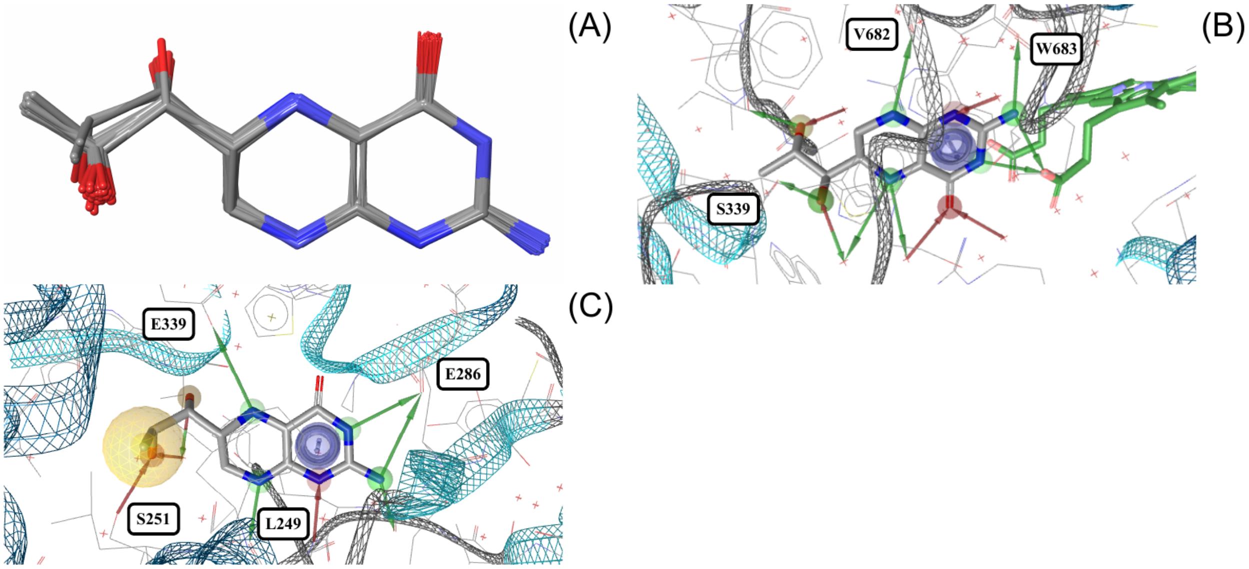

Figure S5. (A) Superposition of 188 high-quality structures of sapropterin. Sapropterin in the binding pocket of (B) human nitric oxide synthase (4D1N, hemoglobin in green) is missing a hydrophobic interaction present in (C) human phenylalanine hydroxylase (1MMK).

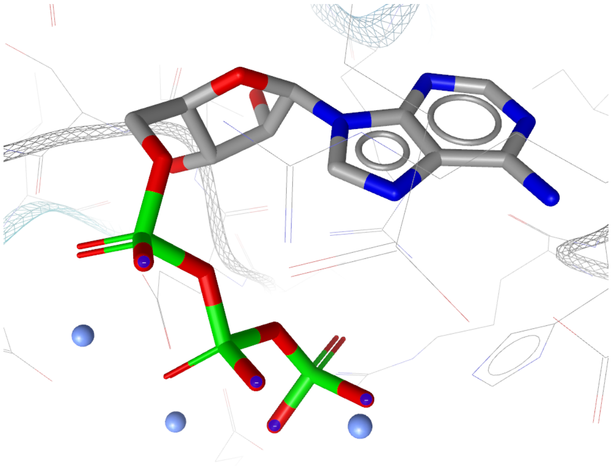

Figure S6. Unusually bent conformer of ATP in the binding pocket of aspartyl-tRNA synthetase from *Pyrococcus kodakaraensis* (1B8A) interacting with three manganese atoms (light blue).

Scheme S1. Overview of all (91) small molecules represented by at least ten conformers in the Sperrylite Dataset.[1]

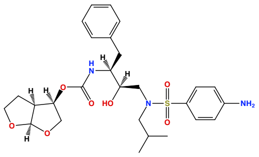<br>017<br>14	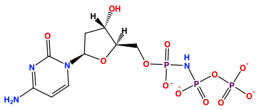<br>0KX<br>11	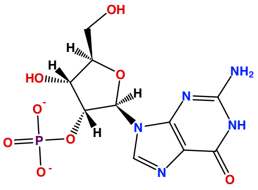<br>2GP<br>14	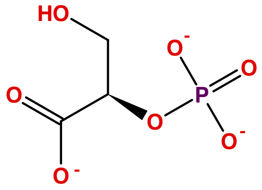<br>2PG<br>10
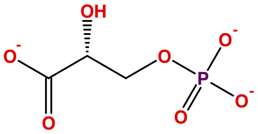<br>3PG<br>23	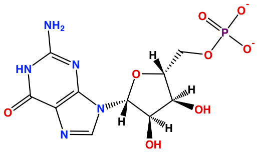<br>5GP<br>31	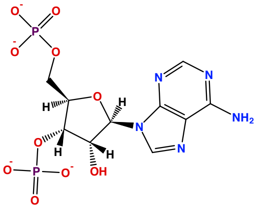<br>A3P<br>29	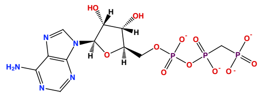<br>ACP<br>29
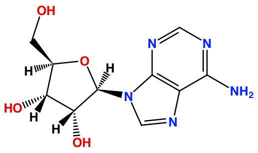<br>ADN<br>57	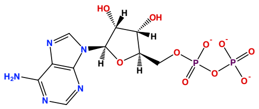<br>ADP<br>462	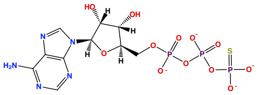<br>AGS<br>17	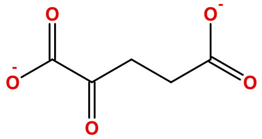<br>AKG<br>77
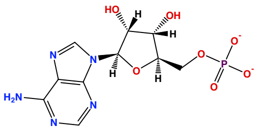<br>AMP<br>171	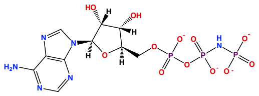<br>ANP<br>140	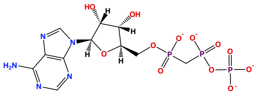<br>APC<br>27	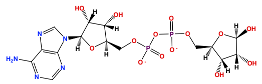<br>APR<br>13
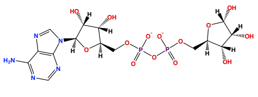<br>AR6<br>15	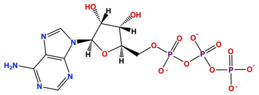<br>ATP<br>212	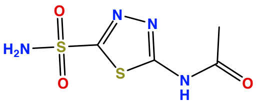<br>AZM<br>10	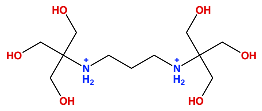<br>B3P<br>22

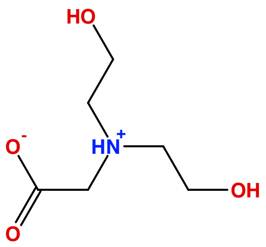<br>BCN<br>13	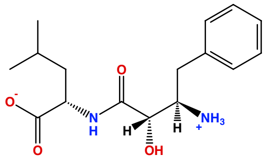<br>BES<br>11	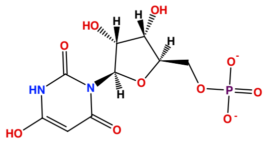<br>BMP<br>29	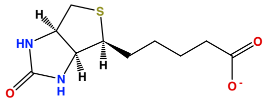<br>BTN<br>43
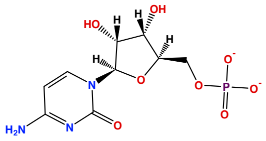<br>C5P<br>30	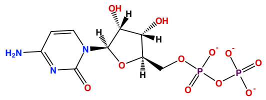<br>CDP<br>17	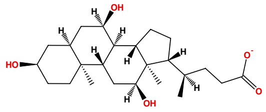<br>CHD<br>13	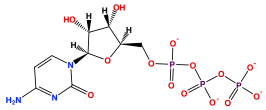<br>CTP<br>19
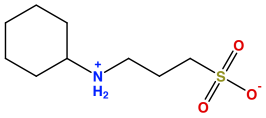<br>CXS<br>18	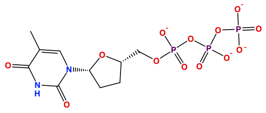<br>D3T<br>10	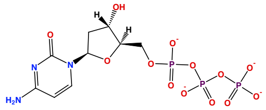<br>DCP<br>39	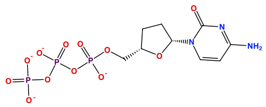<br>DCT<br>11
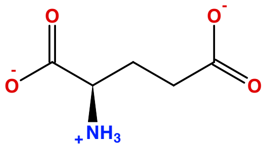<br>DGL<br>14	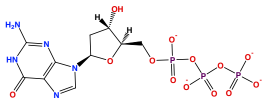<br>DGT<br>28	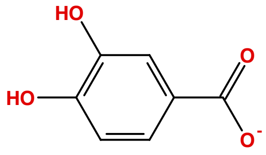<br>DHB<br>10	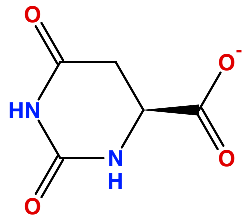<br>DOR<br>15
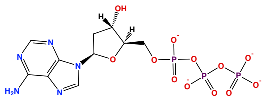<br>DTP<br>33	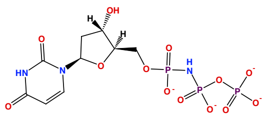<br>DUP<br>26	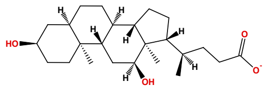<br>DXC<br>18	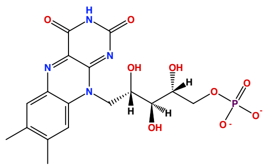<br>FMN<br>370

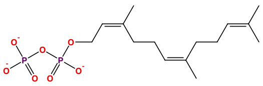<br>FPP<br>15	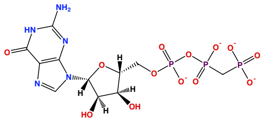<br>GCP<br>14	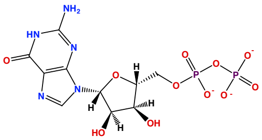<br>GDP<br>261	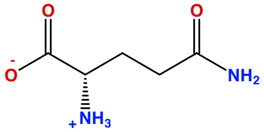<br>GLN<br>16
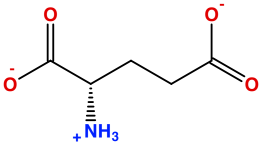<br>GLU<br>113	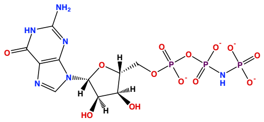<br>GNP<br>139	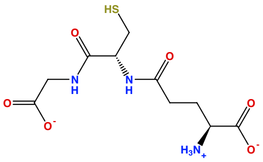<br>GSH<br>74	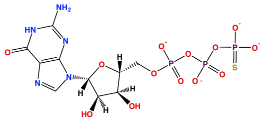<br>GSP<br>20
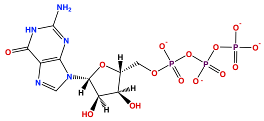<br>GTP<br>96	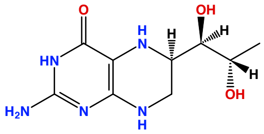<br>H4B<br>188	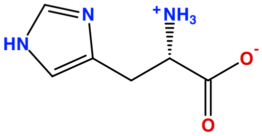<br>HIS<br>28	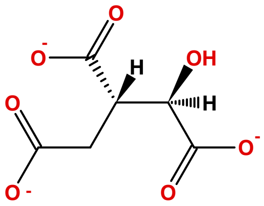<br>ICT<br>10
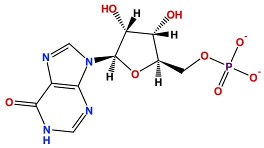<br>IMP<br>26	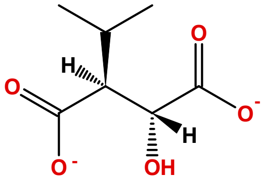<br>IPM<br>14	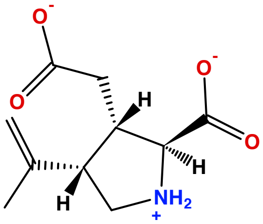<br>KAI<br>10	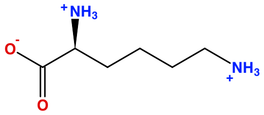<br>LYS<br>18
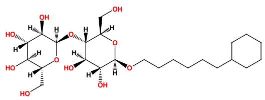<br>MA4<br>18	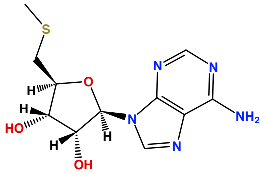<br>MTA<br>24	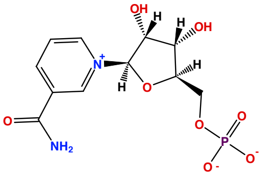<br>NMN<br>13	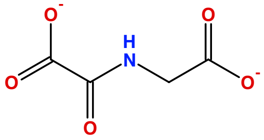<br>OGA<br>44

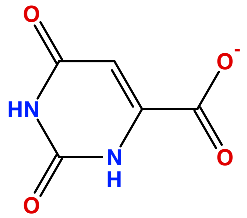

ORO

41

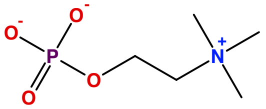

PC

10

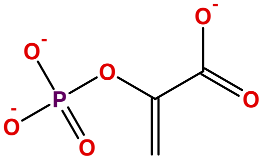

PEP

41

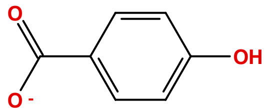

PHB

12

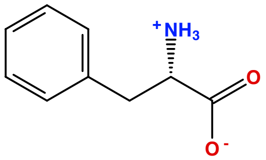

PHE

14

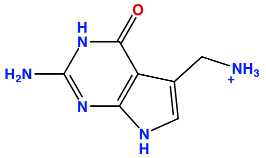

PRF

10

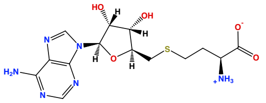

SAH

311

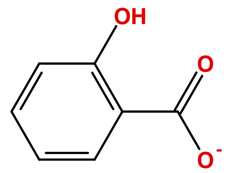

SAL

18

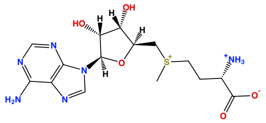

SAM

119

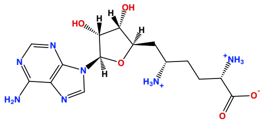

SFG

30

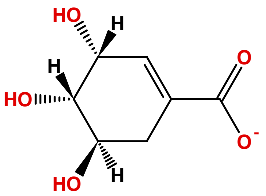

SKM

20

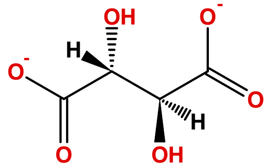

SRT

17

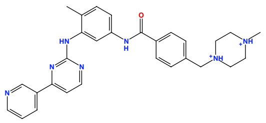

STI

11

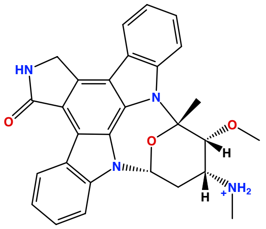

STU

18

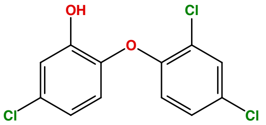

TCL

12

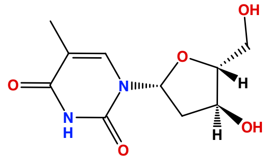

THM

18

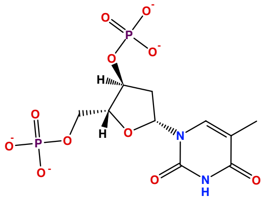

THP

127

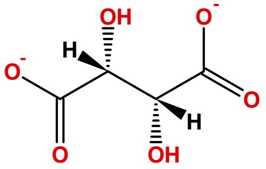

TLA

122

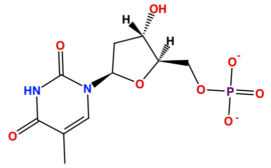

TMP

20

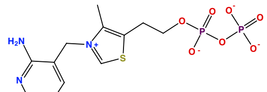

TPP

30

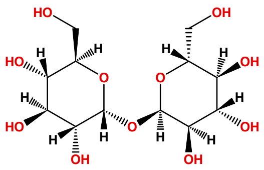<br>TRE<br>27	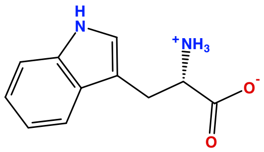<br>TRP<br>22	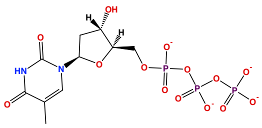<br>TTP<br>30	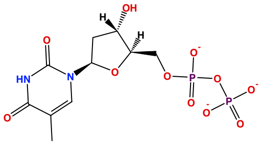<br>TYD<br>14
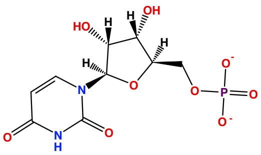<br>U5P<br>21	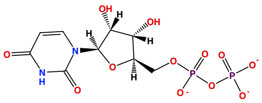<br>UDP<br>102	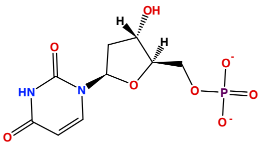<br>UMP<br>41	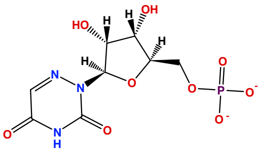<br>UP6<br>10
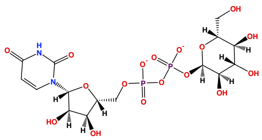<br>UPG<br>20	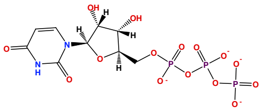<br>UTP<br>14	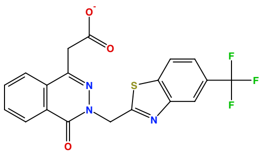<br>ZST<br>11	

[1] For each ligand (specified by the PDB three-letter ligand identifier) the number of conformers in the Sperrylite Dataset is reported.
